# AORI-HAP: a multidimensional risk index to predict in-hospital adverse outcomes in asthma exacerbations

**DOI:** 10.3389/fmed.2025.1707866

**Published:** 2025-12-10

**Authors:** Lishan Yuan, Chongyang Zhao, Lei Wang, Li Zhang, Ying Liu, Lei Liu, Min Feng, Erik Melén, Gang Wang, Shuwen Zhang, Yulai Yuan, Qin Wang, Yilai Li, Deying Kang, Xin Zhang

**Affiliations:** 1 Division of Internal Medicine, Institute of Integrated Traditional Chinese and Western Medicine, West China Hospital, Sichuan University, Chengdu, China; 2 Department of Evidence-based Medicine and Clinical Epidemiology, West China Hospital, Sichuan University, Chengdu, China; 3 NHC Key Laboratory of Clinical Epidemiology and Evidence-based Medicine, West China Hospital, Sichuan University, Chengdu, China; 4 School of Life Sciences, Faculty of Science, University of Technology Sydney, Sydney, NSW, Australia; 5 Respiratory Cellular and Molecular Biology, Woolcock Institute of Medical Research, Sydney, NSW, Australia; 6 Department of Clinical Science and Education, Södersjukhuset, Karolinska Institutet, Stockholm, Sweden; 7 Department of Pulmonary and Critical Care Medicine, Center of Respiratory Medicine, China-Japan Friendship Hospital, Beijing, China; 8 The Department of Respirology of the Affiliated Traditional Chinese Medicine Hospital, Southwest Medical University, Luzhou, China; 9 Department of Clinical Research Management, Center of Biostatistics, Design, Measurement and Evaluation (CBDME), West China Hospital, Sichuan University, Chengdu, China

**Keywords:** asthma exacerbations, AORI-HAP, immune-inflammatory mechanisms, multidimensional risk assessment, predictive scoring system

## Abstract

**Background:**

Despite therapeutic advancements, asthma exacerbations (AEs) remain a major clinical challenge, with immune-inflammatory patterns incompletely characterized. Current guidelines lack robust multidimensional tools for predicting in-hospital adverse outcomes.

**Objective:**

To develop and validate the Asthma Outcome Risk Index for Hospitalized Patients (AORI-HAP), integrating multidimensional predictors, and investigate immune-inflammatory mechanisms underlying adverse outcomes.

**Methods:**

This real-world cohort study enrolled hospitalized AE patients. Univariate analyses identified associations between multidimensional biomarkers and composite outcome (death, ICU admission, invasive ventilation). LASSO logistic regression derived the AORI-HAP, stratifying patients into risk categories. Mediation analysis elucidated mechanistic contributions to adverse outcomes.

**Results:**

The AORI-HAP identified five independent predictors of adverse outcomes: elevated neutrophil-to-lymphocyte ratio (NLR > 8.3, OR = 9.26, *p* < 0.001), increased AST/ALT ratio (>1.41, OR = 3.73, *p* < 0.001), smoking history ≥10 pack-years (OR = 3.54, *p* = 0.005), D-Dimer levels ≥5 mg/L (OR = 3.25, *p* = 0.002), and fasting glucose ≥7 mmol/L (OR = 3.20, *p* = 0.001). Each 3-point increment in the AORI-HAP score corresponded to an additional hospital day (*β* = 0.997, 95% CI: 0.78–1.21, *p* < 0.001), with the model demonstrating strong predictive performance (AUC 0.91, 95% CI 0.86–0.95; sensitivity 90.5%, specificity 69.6%). Mediation analysis revealed that NLR accounted for 26.7% of the total effect linking high-risk status to composite adverse outcome, underscoring its mechanistic relevance.

**Conclusion:**

AORI-HAP facilitates early risk stratification at admission and personalized management in hospitalized asthma patients. NLR’s mediating role underscores its utility as a predictive biomarker and potential therapeutic target.

## Introduction

Asthma exacerbations requiring hospitalization represent critical events in disease management, posing substantial risks of clinical deterioration and mortality (1, 2). These episodes profoundly impair quality of life and drive over 60% of asthma-related healthcare expenditures in high-prevalence regions like the United States, where millions experience annual exacerbations, and Europe, with tens of thousands of asthma-associated deaths reported yearly (3–12). Despite therapeutic advances, in-hospital adverse outcomes—including mortality, ICU admission, and invasive ventilation—persist at concerning rates, underscoring the unmet need for early risk stratification (13, 14).

Multidimensional assessment (MDA) frameworks have transformed prognostication in chronic respiratory diseases (15). For instance, validated tools like the BODE index for COPD and GAP index for ILD integrate clinical, functional, and biochemical parameters to predict mortality and guide therapy (16, 17). Substantial research has been dedicated to examining patients hospitalized for acute exacerbations of ILD and COPD, resulting in specialized prognostic tools to predict outcomes (18–21). These tools comprehensively assess disease severity and prognosis, aiding informed treatment decisions and improved patient management. In stable asthma, the A^2^BCD framework exemplifies multidimensional assessment’s utility for personalized care (22), in this context, the acronym “A^2^BCD” stands for dual Assessment (A^2^) of asthma control and phenotype plus future risk, Basic measures (B) such as education and trigger avoidance, Comorbidities (C) management, and Disease-modifying asthma drugs (D) including phenotype-specific therapies. While guidelines endorse these principles for hospitalized asthma patients, no analogous tool exists for exacerbation management-a critical gap given the multifactorial pathophysiology driving adverse outcomes (2). Existing risk models for asthma exacerbation focus narrowly on isolated factors such as comorbidities or inflammatory biomarkers, neglecting the interplay between clinical, immunological, and metabolic contributors (23–29). These single-dimensional approaches limit prognostic accuracy and fail to identify modifiable therapeutic targets, which highlights the urgent need for developing specialized MDA tools improving clinical care.

We hypothesize that a multidimensional risk index incorporating immune-inflammatory biomarkers and clinical traits can predict in-hospital adverse outcomes in asthma exacerbation patients. Through systematic integration of these predictors, we developed and validated the Asthma Outcome Risk Index for Hospitalized Patients (AORI-HAP). This tool stratifies patients into risk tiers, enabling early identification of high-risk cases and informing precision management strategies aligned with the treatable traits paradigm.

## Methods

### Study design and patients

This prospective cohort study consecutively enrolled adult patients (≥18 years) hospitalized for unplanned asthma exacerbations at West China Hospital, Sichuan University between July 2019 and February 2024. Asthma diagnosis was confirmed through documented airflow variability alongside characteristic respiratory symptoms, as per GINA guidelines (2). Exclusion criteria included misdiagnosis (confirmed by post-hoc specialist review), inability to complete essential tests, hospital stays <24 h, and active COVID-19 infection. To avoid misclassification with COPD and other chronic airway diseases, we excluded patients with a prior COPD diagnosis, a post-bronchodilator FEV₁/FVC below the lower limit of normal, or clinical/imaging findings suggestive of alternative chronic airway pathology. All patients received standardized GINA-based management, as previously detailed (2, 30), including systematic biomarker profiling and daily clinical monitoring until discharge. The cohort was divided into temporally distinct training (July 2019–March 2023; *n* = 1,481) and internal validation (April 2023–February 2024; *n* = 469) sets to, respectively, develop and validate the AORI-HAP model. The study protocol (IRB no. 425) was approved by the hospital’s ethics committee, with written informed consent obtained from all patients.

### Data collection and clinical assessments

Within 24 h of admission, all patients underwent a MDA capturing: (1) demographic/clinical characteristics: age, sex, BMI, smoking status, atopy (positive skin prick test or serum specific IgE > 0.35 kU/L), family asthma history, disease duration, pre-admission medication use, and comorbidities; (2) laboratory profiling: hematologic indices, inflammatory markers, metabolic panels, and cardiac biomarkers; (3) inflammatory ratios: neutrophil-to-lymphocyte ratio (NLR), platelet-to-lymphocyte ratio (PLR), monocyte-to-lymphocyte ratio (MLR), eosinophil-to-lymphocyte ratio (ELR), basophil-to-lymphocyte ratio (BLR), systemic immune-inflammation index (SII), and systemic inflammatory response index (SIRI). These ratios were selected based on their established associations with airway inflammation severity and prognosis in chronic respiratory diseases (31–39).

### Primary and secondary outcomes

The primary composite outcome comprised ICU admission, mechanical ventilation requirement, or all-cause mortality during index hospitalization, reflecting severe exacerbation endpoints (40, 41). Secondary outcomes included individual components of the composite outcome and hospital length of stay (LOS).

### Statistical analysis

Continuous variables were reported as median (interquartile range) and compared using Kruskal-Wallis tests; categorical variables were analyzed with χ^2^ or Fisher’s exact tests. Optimal cutoffs for continuous predictors were determined via receiver operating characteristic (ROC) analysis (Supplementary Table S1). Patients in the training cohort were stratified by composite outcome occurrence for baseline characteristic comparisons (Supplementary Table S2). Univariate logistic regression identified candidate predictors (relative risk [RR] with 95% CI; Supplementary Table S3). To address potential collinearity among inflammatory and metabolic biomarkers, least absolute shrinkage and selection operator (LASSO) regression with an L1 penalty was used for variable selection (Supplementary Figure S1) (42). After model selection, multicollinearity was assessed using the Variance Inflation Factor (VIF) based on a standard logistic regression model. All VIFs were <2, indicating no meaningful collinearity (Supplementary Table S4).

From an initial pool of 56 clinically actionable MDA indicators, 41 demonstrated significant univariate associations with the composite outcome and were entered into the LASSO regression. The LASSO binary logistic regression model was optimized using 10-fold cross-validation, repeated for 2,000 iterations to ensure stability. To balance model performance and simplicity, we applied the ‘one standard error rule’, which selects the most parsimonious model (i.e., the largest *λ* value with the fewest variables) whose cross-validated error is within one standard error of the minimum. This process resulted in a final model containing 13 non-redundant predictors when λ = 0.0135. Given that the primary composite endpoint (death, ICU admission, or invasive ventilation) was a binary outcome (occurrence vs. non-occurrence during hospitalization) rather than a time-to-event variable, we employed binary logistic regression following LASSO feature selection to develop the prediction model. This approach is optimal for early risk stratification at admission, as it estimates the overall probability of an adverse event occurring within the entire in-hospital period based on baseline characteristics. LASSO-derived predictors were weighted by rounded *β*-coefficients to generate the AORI-HAP risk score. Patients were stratified into tertile-based risk groups (low/medium/high) for outcome incidence analysis. Linear regression assessed associations between AORI-HAP scores and LOS.

**Figure 1:**
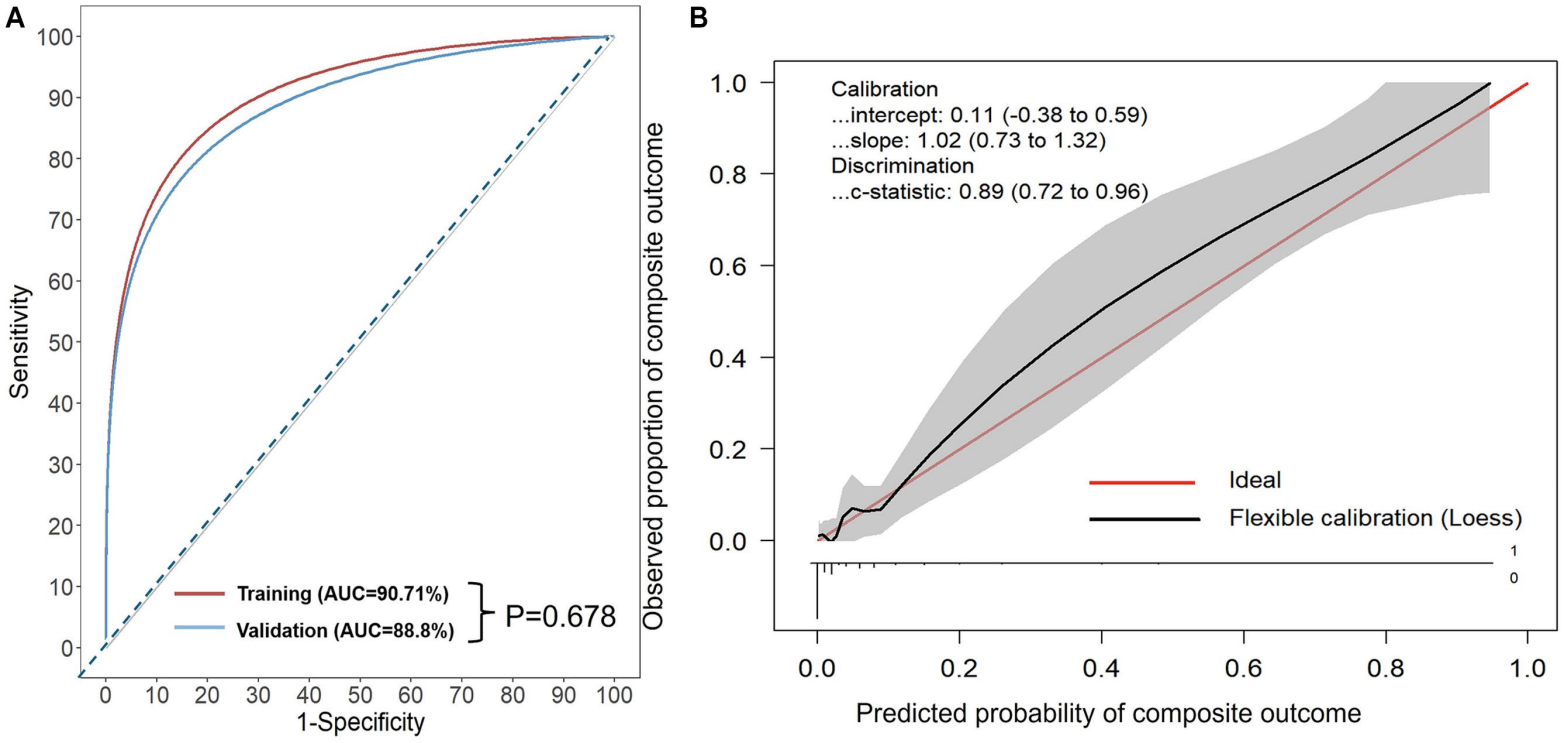
**(A)** Receiver operating characteristic (ROC) curves of the AORI-HAP for predicting the composite outcome. X-axis: represents 1-Specificity, or the false positive rate (FPR). Y-axis: represents the true positive rate (TPR). Area under the curve (AUC): for predicting composite outcome in hospitalized AE patients, the AUC was 0.907 (95% CI: 0.865–0.948) in the training set and 0.888 (95% CI: 0.808–0.967) in the internal validation set. No statistically significant difference was found between the AUCs of the two sets (*p* = 0.678). **(B)** Calibration curves of internal validation set. Calibration-in-the-large represents the difference between the average observed outcomes and the average predicted outcomes. A value of 0 indicates perfect calibration, while values less than or greater than 0 indicate average underestimation or overestimation of the outcome, respectively. The calibration slope, which is the slope of the refitted model, represents the average predictor effects. A value of 1 indicates perfect agreement between the strength of the predictors in the development data and the validation data. The model intercept is 0.11 (95% confidence interval: −0.38 to 0.59) and the slope is 1.02 (95% confidence interval: 0.73 to 1.32), which is close to the ideal value of 1. This indicates that the predicted probabilities by the model have a high degree of consistency with the actual probabilities. The C-statistic is 0.89 (95% confidence interval: 0.72 to 0.96), indicating that the model has high discriminatory power.

Model performance was evaluated in terms of discrimination, calibration, and clinical utility. Discrimination was assessed using the area under the ROC curve (AUC) with 95% confidence intervals, sensitivity, specificity, PPV (positive predictive value), NPV (negative predictive value), PLR (positive likelihood ratio), and NLR (negative likelihood ratio) at various thresholds (Supplementary Table S5) (43). Calibration was examined by calibration slope and intercept as well as calibration plots (Figure 1B). To further assess the clinical utility of the AORI-HAP model, Decision Curve Analysis (DCA) was performed using the rmda package in R, calculating the net benefit across a range of threshold probabilities (0.01–0.50) under a cohort design framework. DCA curves were generated using bootstrap resampling (1,000 iterations) to derive 95% confidence intervals for net benefit estimates. The DCA compared the AORI-HAP model with “treat-all” and “treat-none” strategies in both the training and internal validation cohorts. To evaluate the incremental predictive value of AORI-HAP beyond routine clinical assessment, we constructed a baseline model using variables readily available at admission (age category, sex, smoking history, BMI category, aggregated comorbidity burden, and PaCO₂/PaO₂ categories), following methodological recommendations from TRIPOD and guidance from GINA. Incremental reclassification performance was assessed using category-free NRI and IDI with 2,000 bootstrap replications.

Mediation analysis (R package mediation) quantified pathways linking high-risk AORI-HAP status (reference: low/medium-risk) to composite outcome through inflammatory biomarkers (continuous variables), adjusting for age and sex. Effects were estimated via 1,000 simulations, reporting average causal mediation effects (ACME) and total effects with 95% CIs. Missing data (<3% across laboratory variables) were handled using multiple imputation by chained equations (MICE) under the missing-at-random assumption. All analyses used R v4.3.2 with rms (v6.7.1), ggplot2 (v3.4.4), and shiny (v1.9.0). Statistical significance was defined as two-tailed *p* < 0.05.

## Results

### Demographic and clinical characteristics

**Figure 2:**
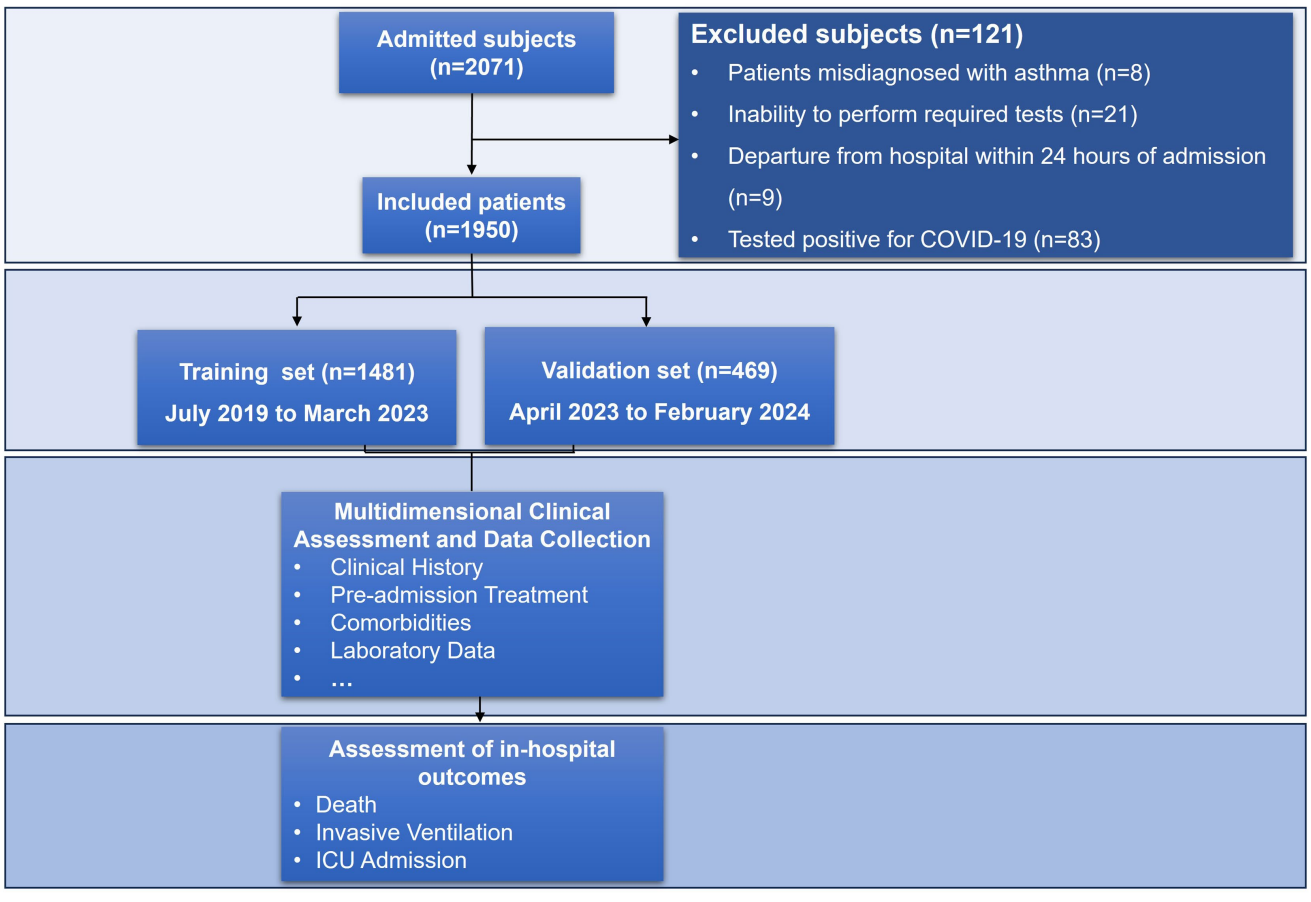
Flowchart of the study.

A total of 1,950 patients hospitalized for acute exacerbation of asthma were included in this study after applying the predefined inclusion and exclusion criteria. The study enrolled 1,481 patients in the training cohort and 469 in the internal validation cohort, with comparable demographic profiles between cohorts (Figure 2; Table 1). In the training cohort, 4.25% (*n* = 63) experienced the composite outcome (ICU admission, mechanical ventilation, or death). Among patients in the training cohort, ICU admission occurred in 17 (0.27%), invasive mechanical ventilation in 59 (3.98%), and in-hospital death in 13 (0.88%); corresponding frequencies in the internal validation cohort were 3 (0.64%), 23 (4.9%), and 6 (1.28%), respectively, and no statistically significant differences were observed between the two cohorts across these outcomes (all *p* > 0.05) (Table 1).

**Table 1:** Comparison of demographic, clinical characteristics, and in-hospital outcomes between the training cohort (*n* = 1,481) and the internal validation cohort (*n* = 469).

Characteristics	Training cohort	Internal validation cohort	*c^2^/H*	*P*
*n*	1,481	469		
Age ≥ 65 years, *n* (%)*	310 (20.93)	98 (20.90)	0.142	0.987
Female, *n* (%)	943 (63.67)	299 (63.75)	0.001	0.975
BMI, kg/m^2^, median (Q1, Q3)	23.08 (20.28, 25.97)	22.39 (19.8,25.48)	3.286	0.070
<18.5, *n* (%)	212 (14.31)	92 (19.62)	8.252	0.051
>28, *n* (%)	164 (11.07)	53 (11.30)	0.001	0.958
Smoking history, ≥ 10 pack-years, *n* (%)	208 (14.04)	77 (16.42)	1.608	0.205
Atopic, *n* (%)	645 (43.55)	211 (44.99)	0.243	0.622
Family history of asthma, *n* (%)	514 (34.70)	169 (36.00)	0.221	0.638
Asthma duration, years, median (Q1, Q3)	6 (2.80, 21.00)	7 (2.00, 20.00)	1.542	0.754
Early-onset asthma, *n* (%)	178 (12.00)	66 (14.00)	1.191	0.275
Prehospital GINA treatment step (4-5), *n* (%)	785 (53.00)	235 (50.00)	1.086	0.297
Prehospital maintenance medications, *n* (%)				
ICS/LABA	843 (56.90)	249 (53)	1.967	0.161
LAMA	60 (4.00)	16 (3.20)	0.237	0.626
Theophylline	205 (13.80)	61 (12.90)	0.146	0.702
Leukotriene modifier	520 (35.10)	175 (37.20)	1.687	0.098
Maintenance OCS	27 (1.80)	7 (1.44)	0.075	0.783
Prehospital OCS use for exacerbations	312 (21.00)	113 (24.00)	1.741	0.187
Comorbidity				
T2 comorbidities, *n* (%)				
Atopic dermatitis	298 (20.12)	97 (20.68)	0.039	0.843
Allergic rhinitis	457 (30.86)	146 (30.13)	0.002	0.957
Nasal polyp	285 (19.24)	93 (19.83)	0.045	0.831
Sinusitis	251 (16.95)	75 (15.99)	0.170	0.679
Non-T_2_ comorbidities, *n* (%)				
Hypertension	270 (18.23)	69 (14.71)	3.071	0.080
Diabetes	186 (12.56)	47 (10.02)	2.180	0.140
CVD	97 (6.55)	33 (7.04)	0.136	0.713
Osteoporosis	45 (3.04)	12 (2.56)	0.289	0.591
GERD	32 (2.16)	4 (0.85)	3.362	0.067
Outcomes, *n* (%)				
Composite outcome#	63 (4.25)	29 (6.18)	2.950	0.086
Invasive ventilation	59 (3.98)	23 (4.90)	0.749	0.387
ICU admission	17 (0.27)	3 (0.64)	0.475	0.491
Death	13 (0.88)	6 (1.28)	0.252	0.616
Hospital LOS, days, median (Q1, Q3)	9 (7, 13)	10 (7, 13)	1.338	0.247

*Data are presented as *n* (%) unless otherwise indicated. Data not normally distributed are presented as median (Q1, Q3). # Consisting of ICU admission, the requirement for invasive ventilation, or all-cause mortality. BMI, body mass index; CVD, cardiovascular disease; GERD, gastroesophageal reflux disease; GINA, global initiative for asthma; ICS, inhaled corticosteroids; ICU, intensive care unit; LABA, long-acting β_2_-agonists; LAMA, long-acting muscarinic antagonist; OCS, oral corticosteroids; LOS, length of stay.

### Inflammatory markers

Patients with composite outcome exhibited pronounced systemic inflammation, characterized by elevated neutrophils (9.71 vs. 4.75 × 10^9^/L), C-reactive protein (CRP) (14.4 vs. 3.56 mg/L), procalcitonin (PCT) (0.13 vs. 0.03 ng/mL), SII (2562.68 vs. 608.42), SIRI (8.31 vs. 1.16) and NLR (17.48 vs. 3.09; all *p* < 0.001), alongside suppressed lymphocytes (0.58 vs. 1.51 × 10^9^/L) and eosinophils (0.10 vs. 1.20 × 10^9^/L; *p* < 0.001) (Supplementary Table S2).

### Multisystem dysfunction

Patients experiencing composite outcome demonstrated multisystem dysfunction beyond inflammatory activation. Nutritional and metabolic derangements were evident, with lower BMI (median 21.30 kg/m^2^, IQR 18.34–23.60; *p* = 0.001) and lower serum albumin levels (37.10 g/L, IQR 32.15–41.30; *p* < 0.001) compared to controls. Concurrently, hyperglycemia (fasting glucose: 7.73 mmol/L, IQR 5.82–10.98; *p* < 0.001) and hepatic stress (AST/ALT ratio: 1.17, IQR 0.75–1.77; *p* < 0.001) were prominent. Cardiac injury markers showed significant elevation, including BNP (B-type natriuretic peptide) (259 pg./mL, IQR 116–979; *p* = 0.001). Coagulopathy was reflected in heightened D-dimer levels (2.11 mg/L, IQR 0.58–24.20; *p* < 0.001), while renal impairment manifested through elevated creatinine (70 μmol/L, IQR 58.50–93.50; *p* = 0.018) and urea (6.44 mmol/L, IQR 5.00–7.92; *p* < 0.001). These findings underscore that adverse outcomes in acute exacerbations arise from synergistic interactions between inflammation, metabolic dysregulation, and multiorgan dysfunction.

### Development and validation of the AORI-HAP

#### Predictive variables in AORI-HAP model development

**Figure 3:**
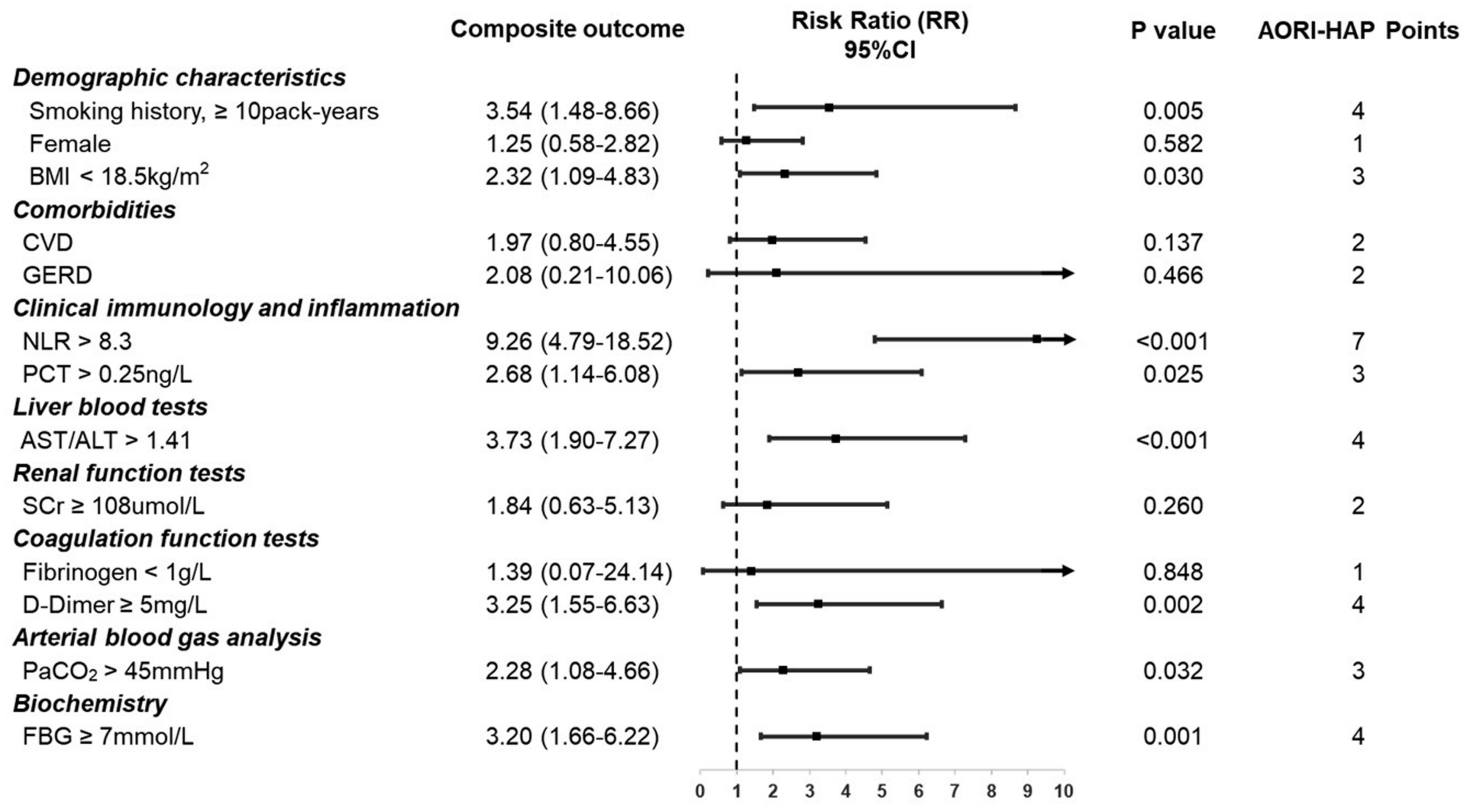
Indicators in multidimensional assessment and associated points in AORI-HAP for predicting composite outcome in hospitalized patients with asthma exacerbations (AE) (*n* = 1,481). Relative risk (RR) represents the strength of the association between each indicator and the composite outcome. AORI-HAP Points: points were assigned based on the indicator’s influence on the composite outcome, to evaluate the patient’s overall risk level. ALT, alanine aminotransferase; AST, aspartate aminotransferase; BMI, body mass index; CVD, cardiovascular disease; FBG, fasting blood glucose; GERD, gastroesophageal reflux disease; NLR, neutrophil-to-lymphocyte ratio; PCT, procalcitonin; PaCO2, partial pressure of carbon dioxide; SCr, serum creatinine.

All 13 predictors selected by LASSO were incorporated into the final AORI-HAP scoring model, with point values assigned proportionally to their *β*-coefficients. The full list of predictors and corresponding AORI-HAP points is provided in Figure 3. The NLR emerged as the strongest predictor, assigned 7 points (RR = 9.26, 95% CI 4.79–18.52; *p* < 0.001). Four variables—smoking history ≥10 pack-years (RR = 3.54, 95% CI 1.48–8.66; *p* = 0.005), AST/ALT ratio >1.41 (RR = 3.73, 95% CI 1.90–7.27; *p* < 0.001), D-dimer ≥5 mg/L (RR = 3.25, 95% CI 1.55–6.63; *p* = 0.002), and FBG ≥ 7 mmol/L (RR = 3.20, 95% CI 1.66–6.22; *p* = 0.001)—each received 4 points. Additional contributors included female sex, underweight status (BMI < 18.5 kg/m^2^), cardiovascular disease (CVD), gastroesophageal reflux disease (GERD), elevated procalcitonin (>0.25 ng/L), renal dysfunction (serum creatinine ≥108 μmol/L), hypofibrinogenemia (<1 g/L), and hypercapnia (PaCO₂ > 45 mmHg).

#### AORI-HAP performance and validation

The AORI-HAP demonstrated excellent discrimination for the composite outcome, achieving an AUC of 0.907 (95% CI: 0.865–0.948) in the training cohort and 0.888 (95% CI: 0.808–0.967) in the internal validation cohort (Figure 1A). At the Youden-optimal threshold (predicted probability = 0.033), the model yielded a sensitivity of 0.873 (95% CI: 0.778–0.952) and a specificity of 0.843 (95% CI: 0.824–0.863) in the training cohort, and 0.880 (95% CI: 0.760–1.000) and 0.802 (95% CI: 0.764–0.838) in the internal validation cohort, respectively. Internal validation revealed minimal overfitting (mean optimism: 0.019). Calibration analysis in the internal validation cohort confirmed strong agreement between predicted and observed risks, with a calibration intercept of 0.11 (95% CI: −0.38 to 0.59) and slope of 1.02 (95% CI: 0.73–1.32), indicating no significant systematic bias or deviation from ideal fit (Figure 1B). DCA demonstrated that the AORI-HAP model provided a higher net clinical benefit than both the “treat-all” and “treat-none” strategies across a broad range of threshold probabilities (0.05–0.45) (Supplementary Figure S5). The training cohort (*n* = 1,481) and internal validation cohort (*n* = 469) showed comparable net benefit curves, supporting the robustness and clinical applicability of the model in identifying patients at high risk for adverse outcomes. Reclassification analyses showed substantial incremental value. In the internal validation cohort, the category-free NRI was 1.258 (95% CI, 0.870–1.559) and the IDI was 0.304 (95% CI, 0.201–0.403). The training cohort demonstrated similarly strong performance, with an NRI of 1.309 (95% CI, 1.084–1.499) and an IDI of 0.311 (95% CI, 0.242–0.380) (Supplementary Table S6). These metrics collectively validate the model’s precision in stratifying in-hospital adverse event risks.

#### Clinical application and accessibility

To enhance clinical translation, we developed an interactive web-based calculator (AORI-HAP Calculator, available at: https://drls.shinyapps.io/AORIHAPapp/) that automates score calculation using real-time inputs. This tool provides instant risk stratification for composite outcome via an intuitive interface, enabling bedside decision support across desktop and mobile platforms without requiring software installation.

### Risk stratification and clinical outcomes by AORI-HAP score

**Figure 4:**
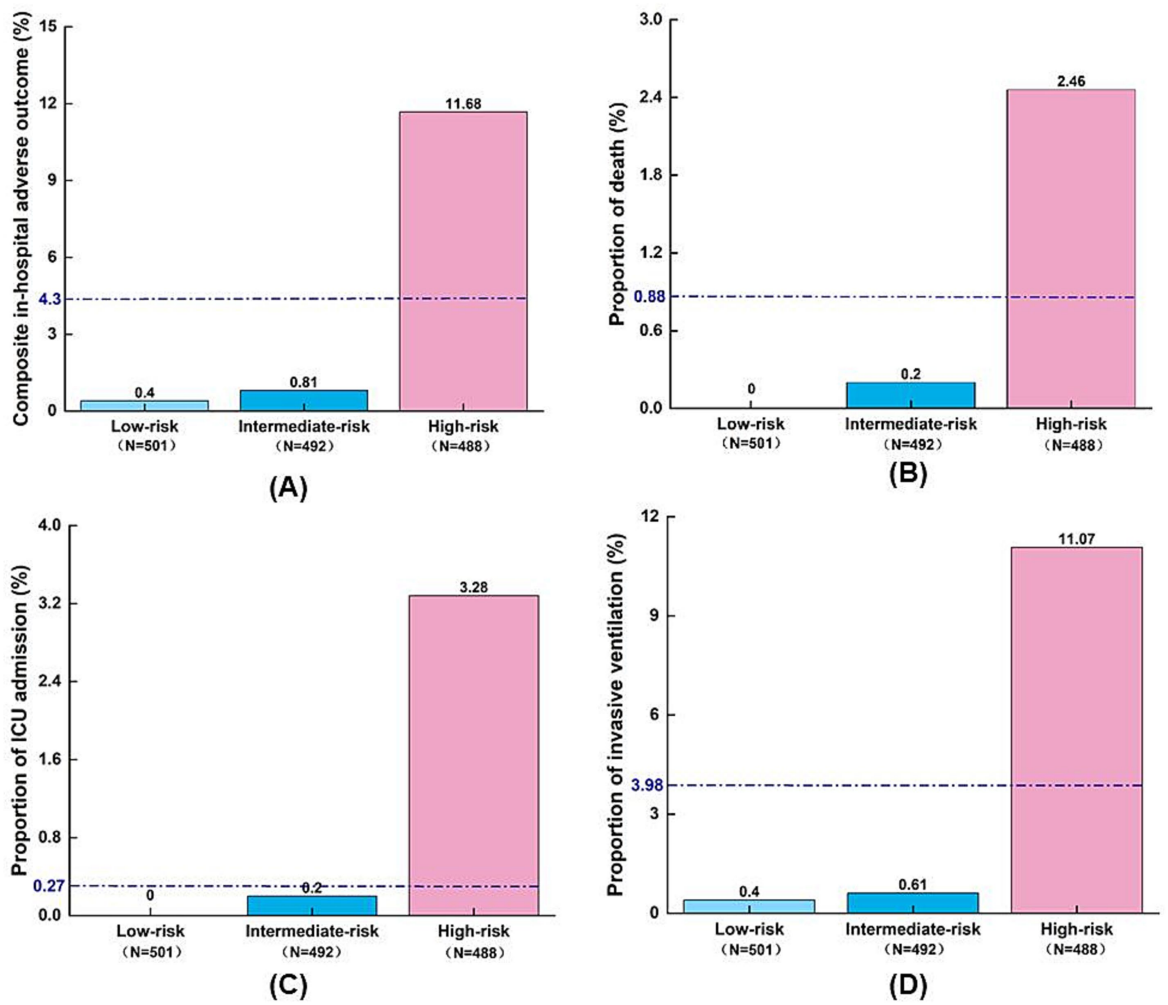
Distribution of adverse outcomes by AORI-HAP risk groups in the training set (*n* = 1,481). **(A)** Composite in-hospital adverse outcome; **(B)** Death; **(C)** ICU admission; **(D)** Invasive ventilation. The blue dashed line indicates the overall incidence of adverse outcomes in the training set population, irrespective of risk group stratification. ICU, intensive care unit.

Risk stratification using AORI-HAP scores in the training cohort (median score: 4, IQR: 1–8) categorized patients into low- (0–1 points, *n* = 501), intermediate- (2–6 points, *n* = 492), and high-risk (≥7 points, *n* = 488) groups (Figure 4). High-risk patients exhibited a 19.3-fold increased incidence of the composite outcome compared to the low/intermediate-risk group (11.68% vs. 0.61%, RR = 19.3, *p* < 0.001), with elevated mortality (2.46% vs. 0.10%, RR = 24.6,), ICU admission (3.28% vs. 0.10%, RR = 30.5), and mechanical ventilation rates (11.07% vs. 0.51%, RR = 21.9; all *p* < 0.001). Hospital stays exceeding the median duration (9 days) progressively increased across risk tiers: 50.4% (low), 57.6% (intermediate), and 66.8% (high; *p* < 0.001; Supplementary Figure S3). The high-risk threshold demonstrated 90.48% sensitivity (95% CI: 83.23–97.72%) and 69.61% specificity (95% CI: 67.21–72.00%) for AORI-HAP prediction (Supplementary Table S4). Internal validation cohort analyses confirmed consistent risk gradient patterns (Supplementary Figure S3).

### Mechanistic pathways linking AORI-HAP to adverse outcomes

**Figure 5:**
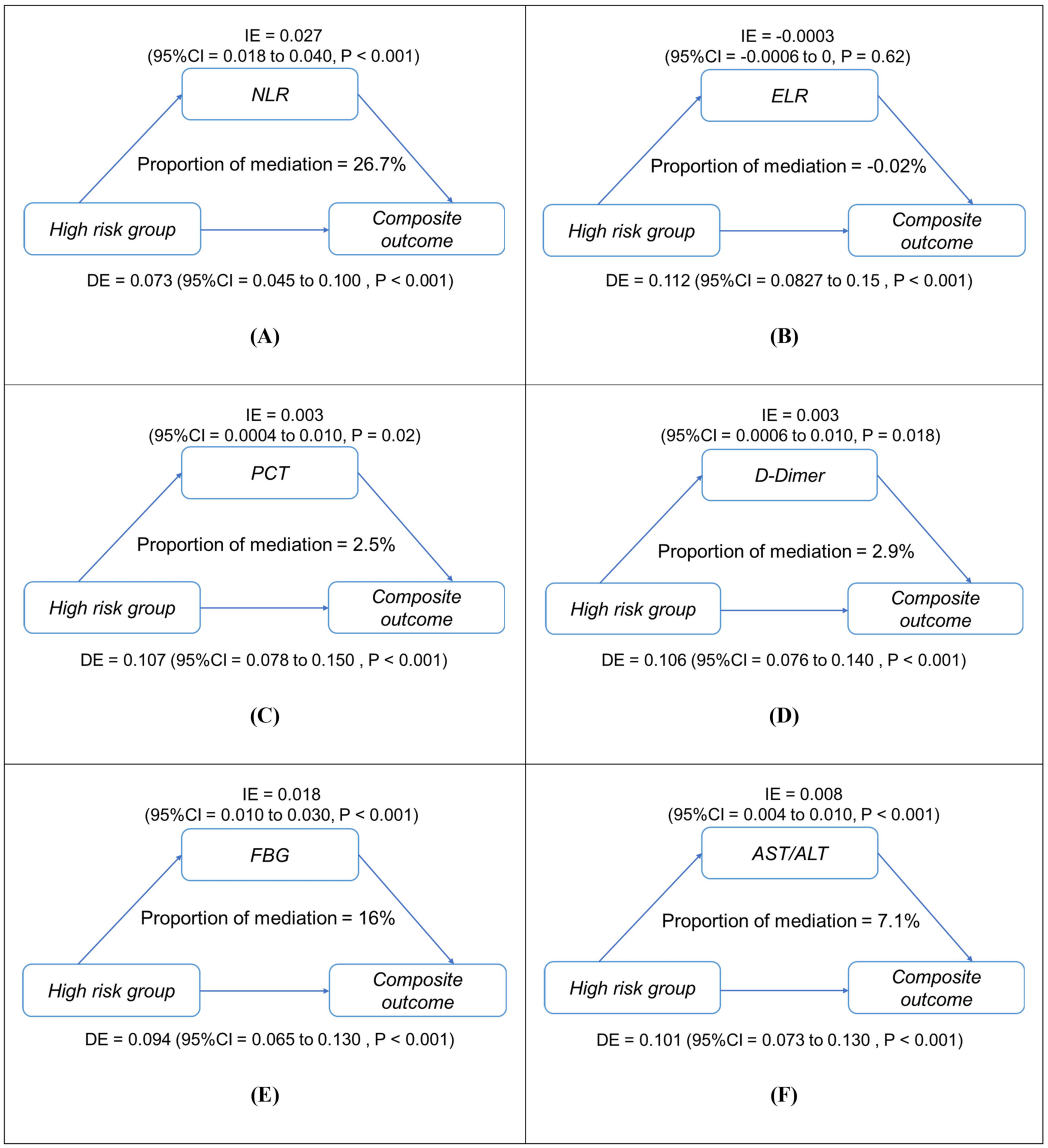
Simplified mediation pathways between high-risk status (reference: low-risk group) and in-hospital composite outcome in patients with asthma exacerbations (AE) (*n* = 1,481). Panels **(A–F)** show mediation models for **(A)** NLR, **(B)** ELR, **(C)** PCT, **(D)** D-dimer, **(E)** FBG, and **(F)** AST/ALT. Each diagram illustrates both the direct effect (DE) of high-risk status on adverse outcomes and the indirect (mediated) effect (IE) transmitted through the mediator, along with their 95% confidence intervals. The proportion mediated (%) represents the share of total effect explained by each mediator. All models were adjusted for age and sex. IE, indirect effect; DE, direct effect; NLR, neutrophil-to-lymphocyte ratio; ELR, eosinophil-to-lymphocyte ratio; PCT, procalcitonin; AST/ALT, aspartate aminotransferase/alanine aminotransferase; FBG, fasting blood glucose. ***p < 0.001, *p < 0.01.

Mediation analysis revealed five biomarkers mediating the association between high-risk status (reference: low-risk) and composite outcome. NLR accounted for 26.7% of the total effect (ACME = 0.027, 95% CI 0.018–0.040; *p* < 0.001), followed by fasting blood glucose (16%, ACME = 0.018), AST/ALT ratio (7.1%), D-dimer (2.9%), and procalcitonin (2.5%) (all *p* < 0.05; Figure 5). NLR demonstrated the strongest pathway-specific effect (*β* = 7.463, *p* < 0.001), with residual direct effects (β = 0.094, *p* < 0.001). Conversely, ELR showed no significant mediation (*p* = 0.62). Sensitivity analyses using medium-risk as the reference group yielded consistent results (Supplementary Figure S4).

## Discussion

We developed the AORI-HAP, a novel prognostic tool integrating 13 routinely measured clinical variables across eight domains. By applying the MDA approach, this model addresses a critical gap in asthma exacerbation research, enabling comprehensive risk stratification through parameters routinely available. The AORI-HAP’s rapid scoring system combines clinical accessibility with prognostic precision, positioning it as a practical tool for guiding in-hospital management and standardizing outcome assessments. Notably, five biomarkers—NLR, PCT, AST/ALT ratio, D-dimer, and FBG—played significant roles as mediators in the relationship between high-risk status and adverse outcomes. Each of these biomarkers demonstrated strong independent predictive value for poor prognoses, underscoring their clinical relevance in identifying high-risk patients. The transformation of continuous variables into binary categories, while enhancing clinical applicability for rapid bedside scoring, represents a methodological compromise that may lead to information loss and potential bias. To mitigate this, cutoff values were rigorously determined using a combination of ROC curve analysis (maximizing Youden’s index), established diagnostic criteria, and hospital reference ranges, ensuring their clinical relevance and statistical justification. Our findings demonstrate that patients experiencing adverse outcomes during asthma exacerbations exhibit a neutrophil-dominant inflammatory phenotype, characterized by neutrophilia alongside lymphopenia. This pattern aligns with acute bacterial infections—a well-established exacerbation trigger (44)—and positions NLR as the strongest prognostic biomarker in the AORI-HAP model, surpassing eosinophil-related indices. While current therapies targeting type 2 inflammation (corticosteroids, biologics) prevent approximately 50% of exacerbations (45, 46), our data emphasize the clinical relevance of neutrophilic inflammation monitoring. Early therapeutic targeting of this subgroup may mitigate adverse outcomes (47).

Patients with immunocompromised status are predisposed to viral infections that may trigger AEs, frequently characterized by lymphopenia and elevated NLR. Our findings demonstrate that NLR significantly mediates the impact of high-risk conditions on composite outcome (31, 48, 49), serving as both a biomarker of disease severity and a prognostic indicator for poorer clinical trajectories. While viral infections remain the predominant clinical trigger for AEs (50, 51), their pathophysiological impact extends beyond direct cytopathic effects to include pro-inflammatory cascades that amplify neutrophil infiltration and degranulation (52), collectively exacerbating airway inflammation and AE severity. Current clinical strategies for AE management emphasize identifying pathogen exposure to optimize therapeutic interventions. Although preliminary studies proposed antiviral therapies (e.g., inhaled IFN-*β*) as potential modulators of antiviral defenses, these approaches lack robust clinical validation (53). Notably, routine viral testing (e.g., PCR) is not standard practice during AE hospitalization, as current evidence fails to establish clear correlations between viral pathogen detection and therapeutic/prognostic outcomes (53). This diagnostic gap parallels the limited clinical utility of antiviral therapies, which have not demonstrated efficacy in reducing AE frequency or severity (54).

While obesity has been associated with adverse outcomes in AE patients (55), our study uniquely identifies low BMI as an independent risk factor for poor prognosis. This finding likely reflects the detrimental impact of malnutrition on immune competence and physiological reserve. These observations underscore the importance of early nutritional risk assessment and appropriate nutritional support as integral components of AE management. Although potential pathways such as adipokine dysregulation and altered energy metabolism have been proposed, these mechanisms were beyond the scope of the current dataset and warrant further investigation in future mechanistic studies (56–66).

While the AST/ALT ratio is conventionally used in hepatology, emerging evidence suggests that it may also reflect systemic inflammation and metabolic stress (67, 68). In our cohort, elevated AST/ALT ratios were observed in 14.4% of patients and independently predicted adverse outcomes even after adjustment for other markers of disease severity (RR_adj_ = 1.31, 95% CI: 1.09–1.58, *p* = 0.0048). Although these findings imply that the AST/ALT ratio may capture systemic and hepatic metabolic disturbances during acute exacerbations (69), further mechanistic and prospective studies are warranted to validate the underlying pathophysiological links. Furthermore, elevated D-dimer levels-established markers of thromboinflammation-emerged as robust prognostic indicators in our cohort, showing significant associations with severe in-hospital outcomes. This elevation likely reflects the systemic inflammatory milieu during AEs, which promotes coagulopathy through endothelial activation and hypercoagulable states (70–72). Notably, such prothrombotic shifts may potentiate microthrombus formation within the bronchial microvasculature, exacerbating airway obstruction and tissue hypoxia (73). The consistent correlation between D-dimer levels and AE severity highlights the clinical value of early coagulation profiling to guide anticoagulation strategies and mitigate thrombotic complications in hospitalized patients.

While AORI-HAP demonstrates clinical promise, several limitations must be acknowledged. First, the model was internally validated using a temporally distinct cohort from the same center, the single-center design inherently limits generalizability, though this aligns with exploratory study paradigms. While external validity requires verification through multicenter trials, the model’s robust discriminatory performance within our cohort (C-statistic 0.89) demonstrates clinical utility for in-hospital risk stratification. Second, although AORI-HAP integrates actionable biomarkers, unmeasured confounders such as dynamic biomarker fluctuations or environmental exposures before admission may influence outcomes. However, the model deliberately prioritized variables routinely captured in clinical workflows, ensuring pragmatic applicability. Third, the focus on short-term outcomes precludes assessment of longitudinal prognosis, yet this aligns with the study’s primary objective to guide acute-phase interventions. Future longitudinal extensions could evaluate sustained treatment effects and exacerbation recurrence. Finally, We acknowledged that induced sputum analysis and spirometry performed during stable phases are important for precise inflammatory phenotyping, but these assessments are not feasible during acute hospital admissions. Therefore, our model relied on routinely collected blood biomarkers—such as eosinophil counts and the neutrophil-to-lymphocyte ratio (NLR)—as practical surrogates of airway inflammation. Future studies should aim to incorporate airway-specific markers when feasible to further refine risk stratification.

## Conclusion

The AORI-HAP constitutes the first multifactorial risk-stratification tool developed to predict adverse in-hospital outcomes in patients hospitalized with AEs. Unlike conventional unidimensional models, AORI-HAP synergistically incorporates MDA profiles to enable risk quantification. This integration facilitates early identification of high-risk patients while supporting personalized precision management. Future studies involving multicenter external validation are warranted to assess the generalizability of the AORI-HAP model across different populations and clinical settings.

## Data Availability

The raw data supporting the conclusions of this article will be made available by the authors, without undue reservation.

## Glossary

ACME: average causal mediation effect
AEs: asthma exacerbations
ALT: alanine aminotransferase
AST: aspartate aminotransferase
AORI-HAP: asthma outcome risk index for hospitalized patients
AUC: area under the curve
BLR: basophil-to-lymphocyte ratio
BMI: body mass index
BNP: B-type natriuretic peptide
CBC: complete blood count
COPD: chronic obstructive pulmonary disease
CRP: C-reactive protein
CVD: cardiovascular disease
ELR: eosinophil-to-lymphocyte ratio
FBG: fasting blood glucose
GERD: gastroesophageal reflux disease
GINA: global initiative for asthma
ICU: intensive care unit
ICS: inhaled corticosteroids
IE: indirect effect
ILD: interstitial lung disease
LABA: long-acting β_2_-agonists
LAMA: long-acting muscarinic antagonist
LASSO: least absolute shrinkage and selection operator
LOS: length of stay
MDA: multidimensional assessment
MLR: monocyte-to-lymphocyte ratio
NLR: neutrophil-to-lymphocyte ratio; negative likelihood ratio;
NPV: negative predictive value
OCS: oral corticosteroids
PaCO_2_: partial pressure of carbon dioxide
PCT: procalcitonin
PPV: positive predictive value
PLR: positive likelihood ratio
PLR: platelet-to-lymphocyte ratio
SCr: serum creatinine
SII: systemic immune-inflammation index
SIRI: systemic inflammatory response index
WBC: leukocytes
