# Supplementary material for: AORI-HAP: a multidimensional risk index to predict in-hospital adverse outcomes in asthma exacerbations
Source: Front Med (Lausanne). 2025 Dec 10;12:1707866. doi: 10.3389/fmed.2025.1707866 (PMC12727603; doi:10.3389/fmed.2025.1707866)
Supplement: Supplementary file 1 [file Supplementary_file_1.docx]

**AORI-HAP: A Multidimensional Risk Index to Predict In-Hospital Adverse Outcomes in Asthma Exacerbations**

**Supplementary appendix**

These supplementary materials provide additional information about the methods and results. The components are as follows:

**Methods**

***Basis for the selection of variable cut-off values***

In determining the cut-off values for each variable, we considered clinical significance, hospital reference ranges, disease diagnostic criteria, from ROC curve analysis, findings from previous studies, and sample characteristics. Additionally, we assessed data quality and engaged in discussion with clinical experts in our hospital to ensure the scientific and clinical relevance of the cut-off values.

Firstly, we referred to the conventional reference ranges provided by the hospital, derived from measurements from a large healthy population. Then, we identified critical threshold values based on the diagnostic criteria for specific diseases. Furthermore, to determine the optimal cut-off values to maximize the sensitivity and specificity of our predictive model, we employed ROC curve analysis. Using the ^1-2^(Youden index = sensitivity + specificity - 1), we identified and selected the cut-off points that maximized this index.

Quantitative variables were transformed into categorical variables based on these cut-off values. Table S1 lists each variable, its corresponding cut-off value, and the source of the cut-off value.

The cut-off values were selected not only for their statistically optimal performance but also for their and relevance in clinical practice. Through these comprehensive methods, we ensured the accuracy of the study results and their practicality for clinical application.

***Definition and calculation of composite inflammatory indices***

Inflammatory biomarkers derived from Complete Blood Count (CBC), such as the Neutrophil-to-Lymphocyte Ratio (NLR), Platelet-to-Lymphocyte Ratio (PLR), Monocyte-to-Lymphocyte Ratio (MLR), Eosinophil-to-Lymphocyte Ratio (ELR), Systemic Immune Inflammation Index (SII), and Systemic Inflammatory Response Index (SIRI), have been used as prognostic factors for various diseases^3-4^. These biomarkers are calculated using specific formulas that incorporate parameters related to neutrophils, lymphocytes, platelets, and monocytes. They provided valuable indicators for assessing the inflammatory and immune status of patients and were utilized in this study to identify risk factors for adverse outcomes. Below are the formulas for calculating each index:

- NLR = (Neutrophil count) / (Lymphocyte count)
- PLR = (Platelet count) / (Lymphocyte count)
- MLR = (Monocyte count) / (Lymphocyte count)
- ELR = (Eosinophil count) / (Lymphocyte count)
- SII = (Platelet count × Neutrophil count) / (Lymphocyte count)
- SIRI = (Neutrophil count × Monocyte count) / (Lymphocyte count)

**Tables**

| Table S1. Clinical index cut-off values and their clinical and statistical Basis* | | | |
| --- | --- | --- | --- |
| Variable class | Variables | Cutoff | Source |
| CBC | WBC | 9.5×10^9^/L | Hospital reference value |
|  | Lymphocytes | 2% |  |
|  |  | [1.1-3.2] ×10^9^/L |  |
|  | Neutrophils | [40, 75] % |  |
|  |  | [1.8, 6.3] ×10^9^/L |  |
|  | Monocytes | [3, 10] % |  |
|  |  | [0.1, 0.6] ×10^9^/L |  |
|  | Eosinophils | [0.4, 8] % |  |
|  |  | [0.1- 0.3] ×10^9^/L |  |
|  | Basophils | 1% |  |
|  |  | 0.6×10^9^/L |  |
| CBC-derived indicators | NLR | 8.3 | ROC curve |
| Nutritional status | BMI | [18.5, 24] kg/m^2^ | BMI standard reference^5-6^ |
|  | Albumin | 35 g/L | Diagnostic criteria for hypoproteinemia^6-7^ |
| Anemic condition | HGB | Male< 130 g/L; Female< 120 g/L | Diagnostic criteria for anemia^8^ |
|  | Severe | <80 g/L |  |
|  | Moderate | 80-109 g/L |  |
|  | Mild | Male 110-129 g/L; Female 110-119 g/L |  |
| Clinical immunology and inflammation | CRP | 50 mg/L | Diagnostic guide for infection^9-10^ |
|  | PCT | 0.25 ng/mL | Guidelines for procalcitonin^11-12^ |
| Lipid-related measures | Cholesterol | 5.2 mmol/L | Diagnostic criteria for hypercholesterolemia^13-14^ |
|  | TG | 1.7 mmol/L | Diagnostic criteria for dyslipidemia^15-16^ |
| Liver blood tests | ALT | 100 IU/L | Diagnostic criteria for hepatic insufficiency^17-18^ |
|  | AST | 80 IU/L |  |
|  | Total Bilirubin | [34.2,51.3] μmol/L | The Child-Pugh score^19^ |
|  | PT | 15.8s |  |
|  | Globulin | [20,40] g/L | Hospital reference value |
|  | AST/ALT | 1.41 | ROC curve |
| Renal function tests | Creatinine | 108 μmol/L | Diagnostic criteria for renal insufficiency^20^ |
|  | Urea | 9.5 mmol/L |  |
| Coagulation function tests | APTT | 43.8s | DIC diagnostic criteria^21^ |
|  | Fibrinogen | 1 g/L |  |
|  | PLT | 100×10^9^/L |  |
|  | D-Dimer | 5mg/L |  |
| Arterial blood gas analysis | PaO_2_ | 60 mmHg | Diagnostic criteria for hypoxemia^22^ |
|  | PaCO_2_ | 45 mmHg | Diagnostic criteria for hypercapnia^23^ |
| Other biochemical indicators | FBG | 7 mmol/L | Diagnostic criteria for diabetes^24^ |
|  | LDH | 250 IU/L | Hospital reference value |
| *Table S1 provides a comprehensive list of the main variables utilized in this study, including their cut-off values and the basis for determining these values. These bases range from the hospital's general reference ranges to specific diagnostic criteria for various diseases, as well as optimal cut-off points obtained through ROC curve analysis. The 'Source' column in the table clarifies how the cut-off values for each variable were determined, ensuring transparency and reproducibility of the study.  ALT, alanine aminotransferase; AST, aspartate aminotransferase; APTT, activated partial thromboplastin time; BMI, body mass index; CBC, complete blood count; CRP, C-reactive protein; FBG, fasting blood glucose; HGB, hemoglobin; LDH, lactate dehydrogenase; NLR, neutrophil-to-lymphocyte ratio; PCT, procalcitonin; PT, prothrombin time; PLT, platelet count; TG, triglyceride; WBC, leukocytes | | | |

| Table S2. Grouping characteristics of adverse outcomes in hospitalized patients with AE | | | | | |
| --- | --- | --- | --- | --- | --- |
| Variables | Total | Non-occurrence | Occurrence | *c^2^*/Z | P value |
| n | 1481 | 1418 | 63 |  |  |
| CBC | | | | | |
| WBC, ×10^9^/L, median (Q1, Q3) | 7.51 (5.62, 9.97) | 7.37 (5.56, 9.79) | 10.56 (8.27, 15.73) | 36.685 | <0.001 |
| >9.5 | 430 (29.03) | 388 (27.36) | 42 (66.67) | 45.225 | <0.001 |
| Lymphocytes, %, median (Q1, Q3) | 21.60 (13.50, 30) | 22.15 (14.30, 30.30) | 5 (2.45, 10.50) | 82.225 | <0.001 |
| <2 | 666 (44.97) | 614 (43.30) | 52 (82.54) | 37.435 | <0.001 |
| Lymphocytes, ×10^9^/L, median (Q1, Q3) | 1.49 (1.04, 1.98) | 1.51 (1.08, 2) | 0.58 (0.33, 1.17) | 74.895 | <0.001 |
| <1.1 | 415 (28.02) | 370 (26.09) | 45 (71.43) | 56.533 | <0.001 |
| Neutrophils, %, median (Q1, Q3) | 69.10 (57.80, 80.60) | 68.50 (57.3, 79.4) | 89.1 (81.20, 94.45) | 71.270 | <0.001 |
| <40 | 34 (2.30) | 33 (2.33) | 1 (1.59) | 57.323 | <0.001 |
| >75 | 536 (36.19) | 484 (34.13) | 52 (82.54) |  |  |
| Neutrophils, ×10^9^/L, median (Q1, Q3) | 4.85 (3.42, 7.18) | 4.75 (3.38, 6.90) | 9.71 (6.50, 14.41) | 56.507 | <0.001 |
| <1.8 | 35 (2.36) | 34 (2.40) | 1 (1.59) | 53.648 | <0.001 |
| >6.3 | 480 (32.41) | 432 (30.47) | 48 (76.19) |  |  |
| Monocytes, %, median (Q1, Q3) | 5.70 (4.20, 7.10) | 5.70 (4.30, 7.10) | 4.1 (2.10, 5.80) | 20.743 | <0.001 |
| <3 | 201 (13.57) | 177 (12.48) | 24 (38.10) | 26.558 | <0.001 |
| >10 | 62 (4.19) | 60 (4.23) | 2 (3.17) |  |  |
| Monocytes, ×10^9^/L, median (Q1, Q3) | 0.40 (0.28, 0.55) | 0.40 (0.28, 0.54) | 0.37 (0.26, 0.56) | 0.075 | 0.784 |
| <0.1 | 71 (4.79) | 69 (4.87) | 2 (3.17) | 1.251 | 0.263 |
| >0.6 | 260 (17.56) | 246 (17.35) | 14 (22.22) |  |  |
| Eosinophils, %, median (Q1, Q3) | 1.10 (0.10, 3.70) | 1.20 (0.10, 3.88) | 0.10 (0.00, 1.15) | 24.903 | <0.001 |
| <0.4 | 513 (34.64) | 472 (33.20) | 41 (65.08) | 25.712 | <0.001 |
| >8 | 164 (11.07) | 162 (11.42) | 2 (3.17) |  |  |
| Eosinophils, ×10^9^/L, median (Q1, Q3) | 0.08 (0.01, 0.24) | 0.08 (0.01, 0.25) | 0.01 (0.00, 0.08) | 27.757 | <0.001 |
| <0.1 | 796 (53.75) | 744 (52.47) | 52 (82.54) | 21.664 | <0.001 |
| >0.3 | 287 (19.38) | 284 (20.03) | 3 (4.76) |  |  |
| Basophils, %, median (Q1, Q3) | 0.20 (0.10, 0.50) | 0.20 (0.10, 0.50) | 0.1 (0.00, 0.20) | 33.007 | <0.001 |
| >1 | 54 (3.65) | 51 (3.60) | 3 (4.76) | 0.019 | 0.889 |
| Basophils, ×10^9^/L, median (Q1, Q3) | 0.02 (0.01, 0.03) | 0.02 (0.01, 0.03) | 0.01 (0.00, 0.02) | 19.479 | <0.001 |
| >0.6 | 89 (6.01) | 85 (5.99) | 4 (6.35) | 0.125 | 0.865 |
| CBC-derived indicators, median (Q1, Q3) | | | | | |
| NLR | 3.16 (1.91, 5.91) | 3.09 (1.90, 5.55) | 17.48 (8.52, 37.77) | 81.532 | <0.001 |
| PLR | 128.37 (89.33, 185.84) | 126.93 (88.66, 181.7) | 260.66 (152.88, 403.14) | 45.764 | <0.001 |
| MLR | 0.26 (0.18, 0.38) | 0.26 (0.18, 0.37) | 0.67 (0.30, 1.30) | 54.109 | <0.001 |
| ELR | 0.05 (0.01, 0.15) | 0.05 (0.01, 0.16) | 0.02 (0.00,0.11) | 9.199 | 0.002 |
| SIRI | 1.19 (0.66, 2.43) | 1.16 (0.66, 2.26) | 8.31 (2.08, 16.06) | 64.063 | <0.001 |
| SII | 626.47(342.96, 1202.8) | 608.42(339.43, 1122.06) | 2562.68(1348.34, 5011.96) | 70.651 | <0.001 |
| Nutritional status | | | | | |
| BMI, kg/m^2^, median (Q1, Q3) | 23.03 (20.17, 25.64) | 23.06 (20.21, 25.76) | 21.30 (18.34, 23.60) | 10.579 | 0.001 |
| <18.5, n (%) | 212 (14.31) | 194 (13.68) | 18 (28.57) | 14.947 | 0.002 |
| >28, n (%) | 164 (11.07) | 160 (11.28) | 4 (6.35) |  |  |
| Albumin, g/L, median (Q1, Q3) | 40.40 (37.40, 43.30) | 40.50 (37.50, 43.30) | 37.10 (32.15, 41.30) | 24.323 | <0.001 |
| <35 | 175 (11.82) | 153 (10.79) | 22 (34.92) | 33.708 | <0.001 |
| Anemic condition | | | | | |
| HGB, g/L, median (Q1, Q3) | 133 (121, 145) | 133 (122, 145) | 122 (106, 145.50) | 9.653 | 0.002 |
| Severe, n (%) | 2 (0.14) | 0 (0.00) | 2 (3.17) | 14.307 | <0.001 |
| Moderate, n (%) | 30 (2.03) | 24 (1.69) | 6 (9.52) |  |  |
| Mild, n (%) | 317 (21.40) | 299 (21.09) | 18 (28.57) |  |  |
| Clinical immunology and inflammation | | | | | |
| CRP, mg/L, median (Q1, Q3) | 3.71 (1.92, 9.04) | 3.56 (1.91, 8.36) | 14.4 (3.59, 32.05) | 28.928 | <0.001 |
| >50 | 85 (5.74) | 73 (5.15) | 12 (19.05) | 19.048 | <0.001 |
| PCT, ng/mL, median (Q1, Q3) | 0.03 (0.02, 0.05) | 0.03 (0.02, 0.05) | 0.13 (0.04, 0.26) | 59.164 | <0.001 |
| >0.25 | 71 (4.79) | 55 (3.88) | 16 (25.40) | 56.569 | <0.001 |
| Lipid-related measures | | | | | |
| Cholesterol, mmol/L, median (Q1, Q3) | 4.43 (3.90, 5.13) | 4.45 (3.94, 5.13) | 4.20 (3.49, 4.76) | 5.842 | 0.016 |
| ≥5.2 | 346 (23.36) | 335 (23.62) | 11 (17.46) | 1.280 | 0.258 |
| TG, mmol/L, median (Q1, Q3) | 1.12 (0.79, 1.58) | 1.12 (0.79, 1.59) | 1.10 (0.74, 1.46) | 0.346 | 0.557 |
| ≥1.7 | 252 (17.02) | 242 (17.07) | 10 (15.87) | 0.061 | 0.805 |
| Liver blood tests | | | | | |
| ALT, IU/L, median (Q1, Q3) | 19 (14,27) | 19 (14,27) | 28 (17,51) | 17.807 | <0.001 |
| >100 | 31 (2.09) | 23 (1.62) | 8 (12.70) | 30.908 | <0.001 |
| AST, IU/L, median (Q1, Q3) | 18 (14,23) | 18 (14,23) | 30 (20, 70) | 49.351 | <0.001 |
| >80 | 27 (1.82) | 14 (0.99) | 13 (20.63) | 119.35 | <0.001 |
| Total Bilirubin, μmol/L, median (Q1, Q3) | 9.5 (7.30, 13) | 9.5 (7.30, 12.80) | 10.4 (7.30, 14.65) | 2.314 | 0.128 |
| <34.2 | 1474 (99.53) | 1412 (99.58) | 62 (98.41) | 1.753 | 0.185 |
| >51.3 | 2 (0.14) | 1 (0.07) | 1 (1.59) |  |  |
| PT, s, median (Q1, Q3) | 11.20 (10.60, 12) | 11.20 (10.60, 11.90) | 11.70 (10.70, 13.15) | 9.333 | 0.002 |
| ≥15.8 | 23 (1.55) | 17 (1.20) | 6 (9.52) | - | <0.001 |
| Globulin, g/L, median (Q1, Q3) | 24.50 (21.90, 27.5) | 24.50 (22, 27.50) | 22.3 (20.20, 27.80) | 3.088 | 0.079 |
| <20 | 173 (11.68) | 161 (11.35) | 12 (19.05) | 2.752 | 0.109 |
| >40 | 10 (0.68) | 9 (0.63) | 1 (1.59) |  |  |
| AST/ALT, median (Q1, Q3) | 0.92 (0.68, 1.25) | 0.91 (0.67, 1.23) | 1.17 (0.75, 1.77) | 14.645 | <0.001 |
| >1.41 | 232 (15.67) | 204 (14.39) | 28 (44.44) | 41.252 | <0.001 |
| Renal function tests | | | | | |
| SCr, μmol/L, median (Q1, Q3) | 66 (56.4, 78) | 66 (56.02, 77.8) | 70 (58.50, 93.50) | 5.548 | 0.018 |
| ≥108 | 55 (3.71) | 45 (3.17) | 10 (15.87) | 23.770 | <0.001 |
| Urea, mmol/L, median (Q1, Q3) | 4.86 (3.80, 6.19) | 4.8 (3.80, 6.07) | 6.44 (5, 7.92) | 25.702 | <0.001 |
| ≥9.5 | 44 (2.97) | 37 (2.61) | 7 (11.11) | 12.319 | <0.001 |
| Coagulation function tests | | | | | |
| APTT, s, median (Q1, Q3) | 26.60 (24.30, 29.40) | 26.60 (24.30, 29.40) | 26.4 (24.25, 29.15) | 0.302 | 0.582 |
| ≥43.8 | 16 (1.08) | 14 (0.99) | 2 (3.17) | - | 0.146 |
| Fibrinogen, g/L, median (Q1, Q3) | 2.86 (2.35, 3.53) | 2.86 (2.35, 3.48) | 2.96 (2.45, 4.21) | 2.382 | 0.123 |
| <1 | 3 (0.20) | 1 (0.07) | 2 (3.17) | - | 0.005 |
| PLT, ×10^9^/L, median (Q1, Q3) | 193 (148, 238) | 194 (149.25, 239) | 159 (121, 187) | 19.500 | <0.001 |
| <100 | 106 (7.16) | 96 (6.77) | 10 (15.87) | 6.214 | 0.013 |
| D-Dimer, mg/L, median (Q1, Q3) | 0.34 (0.20, 0.88) | 0.33 (0.20, 0.83) | 2.11 (0.58, 24.20) | 59.410 | <0.001 |
| ≥5 | 138 (9.32) | 116 (8.18) | 22 (34.92) | 51.044 | <0.001 |
| Arterial blood gas analysis | | | | | |
| PaO_2_, mmHg, median (Q1, Q3) | 84.50 (70.90, 101.90) | 83.55 (70.70, 101.25) | 92.6 (74.90, 120.15) | 4.585 | 0.032 |
| Hypoxemia, n (%) | 137 (9.25) | 135 (9.52) | 2 (3.17) | 2.894 | 0.089 |
| PaCO_2_, mmHg, median (Q1, Q3) | 38 (35.10, 41.90) | 38 (35.10, 41.77) | 38.9 (35.10, 48) | 4.801 | 0.028 |
| Hypercapnia, n (%) | 196 (13.23) | 177 (12.48) | 19 (30.16) | 16.413 | <0.001 |
| Cardiac Biomarkers, median (Q1, Q3) | | | | | |
| TPN-T, ng/L | 7.50 (4.90, 13.20) | 7.50 (4.90, 13.10) | 9.1 (6.70, 29.35) | 5.421 | 0.02 |
| Myoglobin, ng/ml | 28.11 (21, 48.72) | 28.06 (21, 47.65) | 43.63 (25.39, 85.99) | 16.377 | <0.001 |
| CK-MB, ng/ml | 1.63 (1.06, 2.77) | 1.61 (1.06, 2.76) | 2.13 (1.23, 4.53) | 5.951 | 0.015 |
| BNP, ng/L | 150 (55, 375) | 148 (54, 366) | 259 (116, 979) | 10.365 | 0.001 |
| Other biochemical indicators, median (Q1, Q3) | | | | | |
| FBG, mmol/L | 5.09 (4.52, 6.37) | 5.04 (4.51, 6.25) | 7.73 (5.82, 10.98) | 62.369 | <0.001 |
| LDH, IU/L | 173 (150, 205) | 171 (149, 202) | 259 (189, 430.50) | 53.007 | <0.001 |
| >250 | 181 (12.22) | 149 (10.51) | 32 (50.79) | 74.839 | <0.001 |
| Hospital LOS, days, median (Q1, Q3) | 9 (7, 13) | 9 (7, 13) | 13 (9, 20) | 16.238 | <0.001 |
| *Data are presented as n (%) unless otherwise indicated. Data are not normally distributed and presented as median (Q1, Q3).  Anemia was defined as hemoglobin levels below 130 g/L in males and below 120 g/L in females. Hypoxemia was defined as PaO_2_<60 mmHg, and hypercapnia was defined as PaCO_2_>45 mmHg.  ALT, alanine aminotransferase; AST, aspartate aminotransferase; APTT, activated partial thromboplastin time; BMI, body mass index; BLR, basophil-to-lymphocyte ratio; BNP, B-type natriuretic peptide; CBC, complete blood count ; CRP, C-reactive protein; CK-MB, creatine kinase-MB; ELR, eosinophil-to-lymphocyte ratio; FBG, fasting blood glucose; HGB, hemoglobin ; LDH , lactate dehydrogenase; MLR, monocyte-to-lymphocyte ratio; NLR, neutrophil-to-lymphocyte ratio; PLR, platelet-to-lymphocyte ratio; PCT, procalcitonin; PT , prothrombin time; PLT , platelet count; SIRI, systemic inflammatory response index; SII, systemic immune inflammation index; SCr, serum creatinine; TG, triglyceride; TPN-T , troponin T; WBC, leukocytes; LOS, length of stay. | | | | | |

| Table S3. Univariate analysis exploring the associations of the indicators in MDA with the composite outcome. | | |
| --- | --- | --- |
| Variables | RR (95%CI) | P value |
| Age ≥ 65 years | 1.42 (0.78-2.46) | 0.230 |
| Female | 0.61 (0.37-0.94) | 0.048 |
| BMI, kg/m^2^ | 1.01 (0.99-1.02) | 0.286 |
| <18.5 | 1.94 (1.04-3.49) | 0.031 |
| >28 | 0.52 (0.15-1.34) | 0.226 |
| Smoking history, ≥ 10 pack-years | 3.04 (1.72-5.22) | <0.001 |
| Atopic | 1.03 (0.45-2.08) | 0.933 |
| CBC | | |
| WBC, ×10^9^/L | 1.21 (1.15-1.28) | <0.001 |
| >9.5 | 5.31 (3.14-9.24) | <0.001 |
| Lymphocytes, % | 0.84 (0.80-0.87) | <0.001 |
| <20 | 6.11 (3.29-12.44) | <0.001 |
| Lymphocytes, ×10^9^/L | 0.09 (0.05-0.16) | <0.001 |
| <1.1 | 7.2 (4.15-13.09) | <0.001 |
| >3.2 | 1.44 (0.08-7.30) | 0.724 |
| Neutrophils, % | 1.11 (1.08-1.14) | <0.001 |
| <40 | 2.73 (0.15-14.89) | 0.345 |
| >75 | 9.68 (5.09-20.37) | <0.001 |
| Neutrophils, ×10^9^/L | 1.32 (1.24-1.41) | <0.001 |
| <1.8 | 2.00 (0.11-10.40) | 0.509 |
| >6.3 | 7.56 (4.23-14.36) | <0.001 |
| Monocytes, % | 0.78 (0.69-0.87) | <0.001 |
| <3 | 4.33 (2.50-7.36) | <0.001 |
| >10 | 1.06 (0.17-3.59) | 0.933 |
| Monocytes, ×10^9^/L | 1.57 (0.53-4.27) | 0.397 |
| <0.1 | 0.68 (0.11-2.26) | 0.599 |
| >0.6 | 1.34 (0.70-2.40) | 0.355 |
| Eosinophils, % | 0.91 (0.83-0.99) | 0.045 |
| <0.4 | 3.41 (2-5.99) | <0.001 |
| >8 | 0.48 (0.08-1.68) | 0.331 |
| Eosinophils, ×10^9^/L | 0.03 (0.01-0.10) | 0.001 |
| <0.1 | 3.41 (1.70-7.82) | 0.001 |
| >0.3 | 0.51 (0.11-1.8) | 0.330 |
| Basophils, % | 0.18 (0.05-0.52) | 0.004 |
| >1 | 1.34(0.32-3.78) | 0.630 |
| Basophils, ×10^9^/L | 0.04 (0.01-0.09) | 0.024 |
| >0.6 | 1.06 (0.32-2.66) | 0.908 |
| HGB, g/L | 0.97 (0.96-0.98) | <0.001 |
| Anemic | 2.66 (1.57-4.45) | <0.001 |
| Clinical immunology and inflammation | |  |
| CRP, mg/L | 1.01 (1.01-1.02) | <0.001 |
| >50 | 4.34 (2.13-8.24) | <0.001 |
| PCT, ng/mL | 1.15 (1.07-1.26) | <0.001 |
| >0.25 | 8.44 (4.40-15.56) | <0.001 |
| Lipid-related measures | | |
| Cholesterol, mmol/L | 0.70 (0.53-0.91) | 0.010 |
| ≥5.2 | 0.68 (0.34-1.28) | 0.261 |
| TG, mmol/L | 0.95 (0.68-1.25) | 0.733 |
| ≥1.84 | 0.92 (0.43-1.75) | 0.805 |
| Liver blood tests | | |
| ALT, IU/L | 1.01 (1.01-1.08) | <0.001 |
| >100 | 8.82 (3.56-19.88) | <0.001 |
| AST, IU/L | 1.02 (1.02-1.03) | <0.001 |
| >80 | 26.07 (11.54-58.71) | <0.001 |
| Total Bilirubin | 1.04 (1.01-1.07) | 0.016 |
| >51.3, umol/L | 5.85 (0.23-2.39) | 0.981 |
| PT, s | 1.08 (1.02-1.15) | 0.005 |
| ≥15.8 | 8.67 (3.04-21.77) | <0.001 |
| Albumin, g/L | 0.88 (0.84-0.92) | <0.001 |
| <35 | 4.44 (2.54-7.58) | <0.001 |
| Globulin, g/L | 0.97 (0.91-1.02) | 0.273 |
| <20 | 1.86 (0.93-3.45) | 0.062 |
| >40 | 2.77 (0.15-15.18) | 0.338 |
| AST/ALT | 2.39 (1.71-3.35) | <0.001 |
| >1.41 | 4.76 (2.82-7.98) | <0.001 |
| Renal function tests |  |  |
| SCr, umol/L | 1.01 (1-1.01) | 0.001 |
| ≥108 | 5.76 (2.62-11.63) | <0.001 |
| Urea, mmol/L | 1.24 (1.15-1.34) | <0.001 |
| ≥9.5 | 4.67 (1.84-10.34) | <0.001 |
| Coagulation and cardiovascular function | | |
| APTT, s | 1.00 (0.94-1.04) | 0.873 |
| ≥43.8 | 3.29 (0.51-12.12) | 0.121 |
| Fibrinogen<1 g/L | 4.46 (4.39-10.81) | 0.002 |
| PLT, ×10^9^/L | 0.93 (0.42-1.57) | <0.001 |
| <100 | 2.44 (1.14-4.75) | 0.014 |
| D-Dimer ≥5mg/L | 6.02 (3.42-10.36) | <0.001 |
| TPN-T, ng/L | 1.02 (1.01-1.02) | <0.001 |
| CK-MB, ng/ml | 1.01 (0.99-1.02) | 0.235 |
| Arterial blood gas analysis | |  |
| PaO_2_, mmHg | 1.01 (0.04-1.07) | <0.001 |
| Hypoxemia | 0.31 (0.05-1.01) | 0.107 |
| PaCO_2_, mmHg | 1.05 (1.03-1.07) | <0.001 |
| Hypercapnia | 3.03 (1.69-5.23) | <0.001 |
| CBC-derived indicators | | |
| NLR | 1.13 (1.11-1.16) | <0.001 |
| PLR | 1.87 (1.03-1.01) | <0.001 |
| MLR | 21.41 (11.56-41.39) | <0.001 |
| ELR | 0.44 (0.08-1.06) | 0.247 |
| BLR | 8.14 (2.43-10.49) | 0.001 |
| SIRI | 1.31 (1.24-1.37) | <0.001 |
| SII | 1.21 (1.43-1.56) | <0.001 |
| Other biochemical indicators | | |
| LDH, IU/L | 1.01 (0.03-1.06) | <0.001 |
| >250 | 8.73 (5.15-14.83) | <0.001 |
| FBG, mmol/L | 1.29 (1.21-1.37) | <0.001 |
| T_2_ comorbidities | | |
| Atopic dermatitis | 0.65 (0.31-1.54) | 0.979 |
| Allergic rhinitis | 0.32 (0.21-0.52) | 0.980 |
| Nasal polyp | 0.54 (0.41-0.62) | 0.985 |
| Sinusitis | 1.11 (0.27-3.12) | 0.861 |
| Non-T_2_ comorbidities | | |
| Hypertension | 1.17 (0.6-2.13) | 0.614 |
| Diabetes | 1.17 (0.53-2.29) | 0.673 |
| CVD | 3.69 (1.82-6.97) | <0.001 |
| Osteoporosis | 0.50 (0.03-2.37) | 0.501 |
| GERD | 0.72 (0.04-3.45) | 0.040 |
| Anemia was defined as hemoglobin levels below 130 g/L in males and below 120 g/L in females. Hypoxemia was defined as PaO_2_<60 mmHg, and hypercapnia was defined as PaCO_2_>45 mmHg.  ALT, alanine aminotransferase; AST, aspartate aminotransferase; APTT, activated partial thromboplastin time; AISI, aggregate index of systemic inflammation; BMI, body mass index; BLR, basophil-to-lymphocyte ratio; CBC, complete blood count; CRP, C-reactive protein; CVD, cardiovascular disease; CK-MB, creatine kinase-MB; ELR, eosinophil-to-lymphocyte ratio; FBG, fasting blood glucose; GERD, gastroesophageal reflux; HGB, hemoglobin; LDH, lactate dehydrogenase; MLR, monocyte-to-lymphocyte ratio; NLR, neutrophil-to-lymphocyte ratio; PCT, procalcitonin; PT, prothrombin time; PLT, platelet count; PLR, platelet-to-lymphocyte ratio; SIRI, systemic inflammatory response index; SII, systemic immune inflammation index; SCr, serum creatinine ; TPN-T, troponin T; TG, triglyceride; WBC, leukocytes. | | |

| Table S4. Sensitivity and specificity values for AORI-HAP | | | | | | |
| --- | --- | --- | --- | --- | --- | --- |
|  | PLR (95%CI) | NLR (95%CI) | Sensitivity (95%CI) | Specificity (95%CI) | PPV (95%CI) | NPV (95%CI) |
| Training set | | | | | | |
| Low-risk and intermediate-risk group vs. high-risk group | 2.98 (2.31-3.83) | 0.14 (0.06-0.32) | 90.48 (83.23-97.72) | 69.61 (67.21-72.00) | 11.68 (8.83-14.53) | 99.4 (98.91-99.88) |
| Intermediate-risk and high-risk group vs. low-risk group | 1.49 (1.15-1.93) | 0.09 (0.02-0.37) | 96.83 (92.50-100.00) | 35.19 (32.70-37.68) | 6.22 (4.71-7.74) | 99.6 (99.05-100.00) |
| Validation set | | | | | | |
| Low-risk and intermediate-risk group vs. high-risk group | 2.59 (1.77-3.79) | 0.16 (0.05-0.55) | 89.29 (77.83-99.32) | 65.53 (61.10-69.97) | 14.12 (8.99-19.26) | 98.97 (97.82-99.96) |
| Intermediate-risk and High-risk group vs. low-risk group | 1.33 (0.89-2.00) | 0.24 (0.06-1.01) | 92.86 (83.32-100.00) | 30.16 (25.88-34.44) | 7.78 (4.91-10.66) | 98.52 (96.48-99.98) |
| AORI-HAP: adverse outcomes risk index for hospitalized asthma patients IV: invasive ventilation; NLR: negative likelihood ratio; NPV: negative predictive value; PPV: positive predictive value; PLR: positive likelihood ratio; | | | | | | |

| Table S5. Variance Inflation Factors (VIF) for predictors in the final logistic regression model (training cohort). | | |
| --- | --- | --- |
| Predictor | Definition (coding) | VIF |
| Smoking history | ≥10 pack-years = 1, otherwise = 0 | 1.65 |
| Sex | Female = 1, Male = 0 | 1.65 |
| BMI | <18.5 kg/m² = 1, ≥18.5 = 0 | 1.08 |
| Comorbidities |  |  |
| CAD | Presence of cardiovascular disease = 1, No = 0 | 1.12 |
| GERD | Diagnosed GERD = 1, No = 0 | 1.02 |
| Clinical immunology and inflammation |  |  |
| NLR | >8.3 = 1, ≤8.3 = 0 | 1.13 |
| PCT | >0.25 ng/mL = 1, ≤0.25 = 0 | 1.05 |
| Liver blood tests |  |  |
| AST/ALT | >1.41 = 1, ≤1.41 = 0 | 1.11 |
| Renal function tests |  |  |
| SCr | ≥108 µmol/L = 1, <108 = 0 | 1.19 |
| Coagulation function tests |  |  |
| Fibrinogen | ≥5 mg/L = 1, <5 = 0 | 1.01 |
| D-Dimer | ≥5 mg/L = 1, <5 = 0 | 1.02 |
| Arterial blood gas analysis |  |  |
| PaCO_2_ | >45 mmHg = 1, ≤45 = 0 | 1.04 |
| Biochemistry |  |  |
| FBG | ≥7 mmol/L = 1, <7 = 0 | 1.18 |
| ALT, alanine aminotransferase; AST, aspartate aminotransferase; BMI, body mass index; CVD, cardiovascular disease; FBG, fasting blood glucose; GERD, gastroesophageal reflux disease; NLR, neutrophil-to-lymphocyte ratio; PCT, procalcitonin; PaCO2, partial pressure of carbon dioxide; SCr, serum creatinine.  All continuous biomarkers were dichotomized according to clinically relevant cutoffs or optimal Youden index values. VIF values were calculated using standard logistic regression fitted on the training cohort; GVIFs were converted to equivalent VIFs as GVIF^(1/(2×Df)). All VIF values < 2 indicate absence of meaningful multicollinearity. | | |

| Table S6. Reclassification Statistics for the AORI-HAP Model | | | | |
| --- | --- | --- | --- | --- |
| Cohort | Continuous NRI | 95% CI | IDI | 95% CI |
| Training cohort | 1.309 | 1.084–1.499 | 0.311 | 0.242–0.380 |
| Internal validation cohort | 1.258 | 0.870–1.559 | 0.304 | 0.201–0.403 |
| NRI, net reclassification improvement, IDI, integrated discrimination improvement, CI, confidence interval. The baseline model included age category, sex, smoking history, BMI category, aggregated comorbidity burden, PaCO₂ category, and PaO₂ category. The extended model incorporated the AORI-HAP score as an additional predictor. Continuous NRI and IDI were calculated using 2,000 bootstrap replications. | | | | |

**Figures**


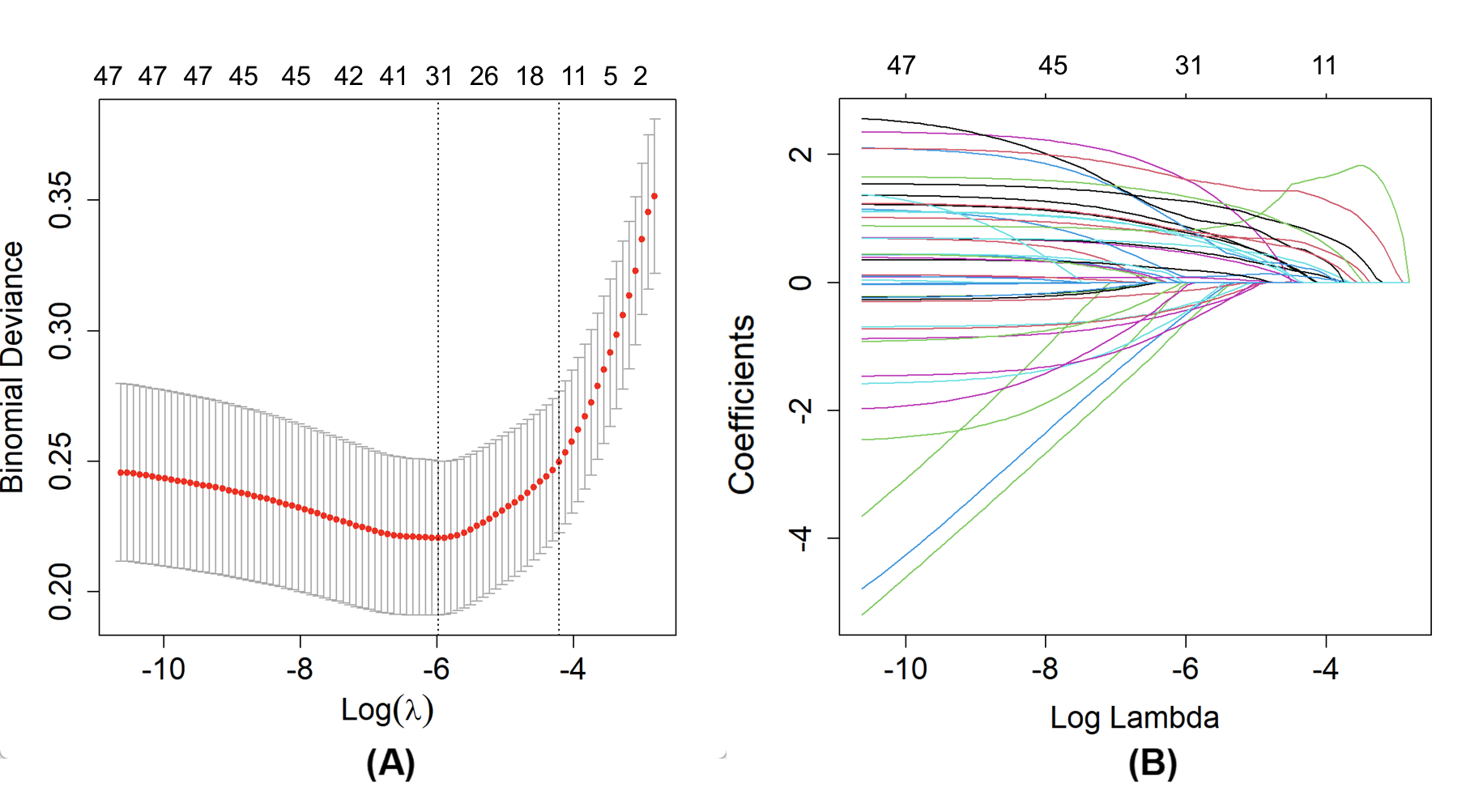


Figure S1. Predictor selection using the least absolute shrinkage and selection operator (LASSO) binary logistic regression model

(A) The selection of the tuning parameter (λ) in the LASSO model using the 10-fold cross-validation procedure is plotted as a function of log (λ). The y-axis represents partial likelihood deviance, and the lower x-axis represents log (λ). Numbers along the upper x-axis represent the average number of predictors. The red dots indicate the average deviance values for each model with a given λ, where the model provides its best fit to data. The dotted vertical lines are drawn at two special values along the λ sequence. The left vertical line uses the value of λ that gives the minimum mean cross-validated error. The right vertical line uses the value of λ that gives the most regularized model such that the cross-validated error is within one standard error of the minimum. The 10-fold cross-validation method was applied to the iterative analysis, resulting in a model with excellent performance but a minimum number of variables when λ was 0.0135 (Log λ= -4.30).

(B) LASSO coefficient profiles of the 47 baseline features. Each curve corresponds to a variable. A coefficient profile plot is generated against the log (λ) sequence. LASSO, least absolute shrinkage and selection operator; SE, standard error.

| 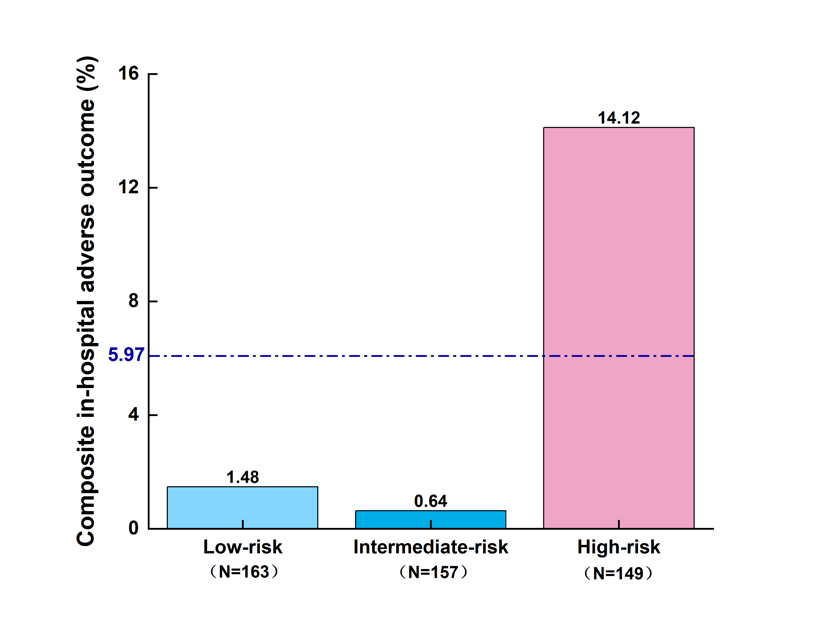**(A)** | 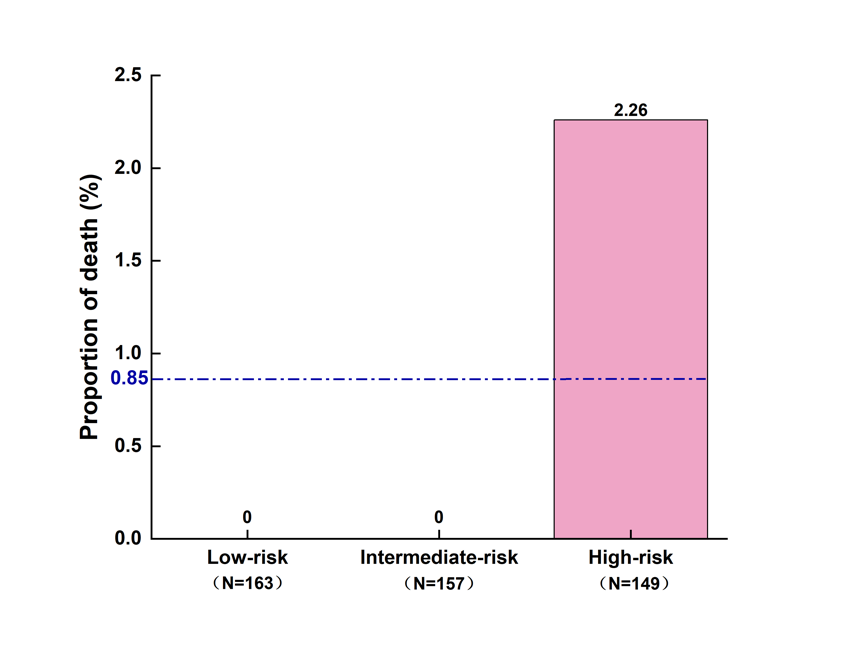**(B)** |
| --- | --- |
| 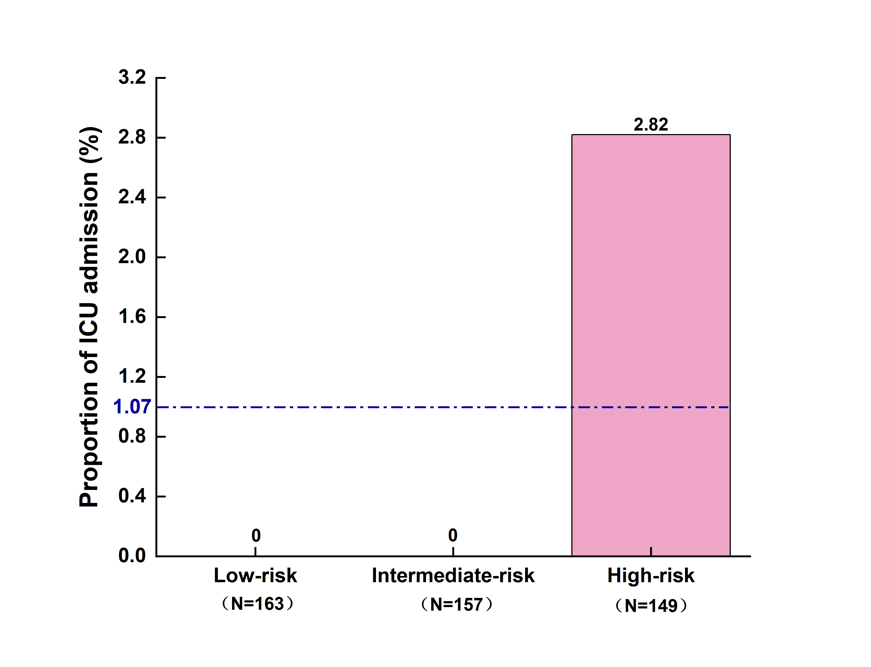**(C)** | 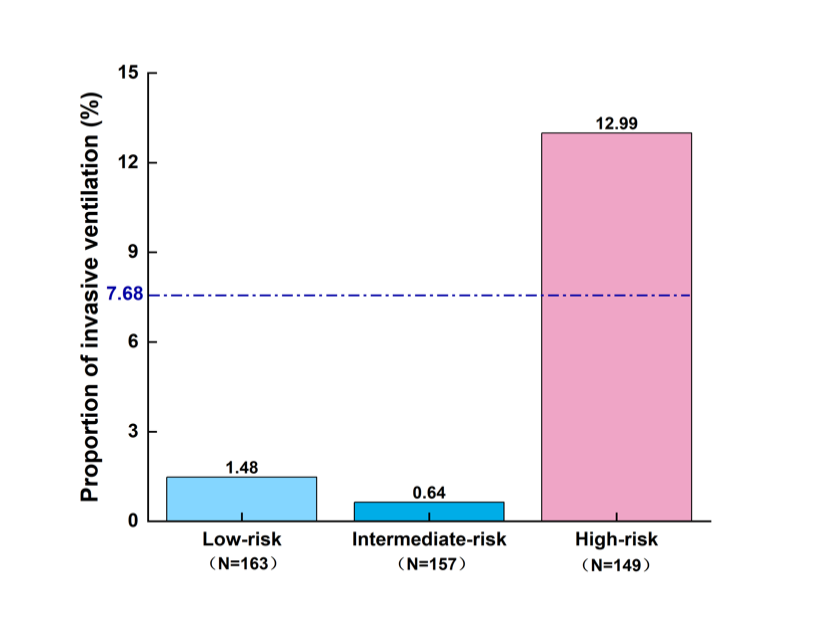**(D)** |

Figure S2. Distribution of adverse outcomes across AORI-HAP risk groups within the validation set. (A) Composite in-hospital adverse outcome; (B) Death; (C) ICU admission; (D) Invasive ventilation. The blue dashed line indicates the overall incidence of adverse outcomes in the training set population, irrespective of risk group stratification. ICU, intensive care unit. ICU, intensive care unit; IV: invasive ventilation.


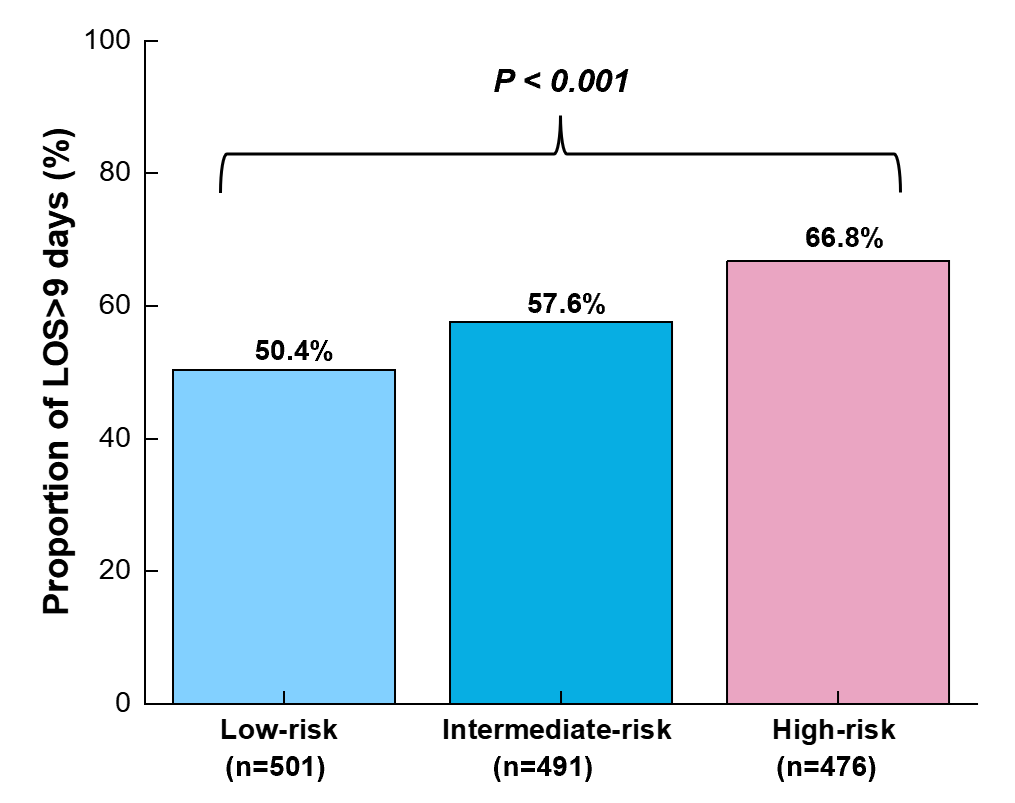


Figure S3. Proportions of patients with hospital stays exceeding the median duration of 9 days, categorized by AORI-HAP risk groups. To reduce bias in the duration of hospital stays caused by deceased patients, the analysis was restricted to those who were alive at the end of the observation period. LOS refers to length of stay.

Figure S4. Simplified mediation pathways between high-risk status (reference: medium- and low-risk group) and in-hospital composite outcomes in patients with asthma exacerbations (AE) (n = 1,481). Panels (A–F) show mediation models for (A) NLR, (B) ELR, (C) PCT, (D) D-dimer, (E) FBG, and (F) AST/ALT. Each diagram illustrates both the direct effect (DE) of high-risk status on adverse outcomes and the indirect (mediated) effect (IE) transmitted through the mediator, along with their 95% confidence intervals. The proportion mediated (%) represents the share of total effect explained by each mediator. All models were adjusted for age and sex. IE, indirect effect; DE, direct effect; NLR, neutrophil-to-lymphocyte ratio; ELR, eosinophil-to-lymphocyte ratio; PCT, procalcitonin; AST/ALT, aspartate aminotransferase/alanine aminotransferase; FBG, fasting blood glucose. ***P < 0.001, *P < 0.01.

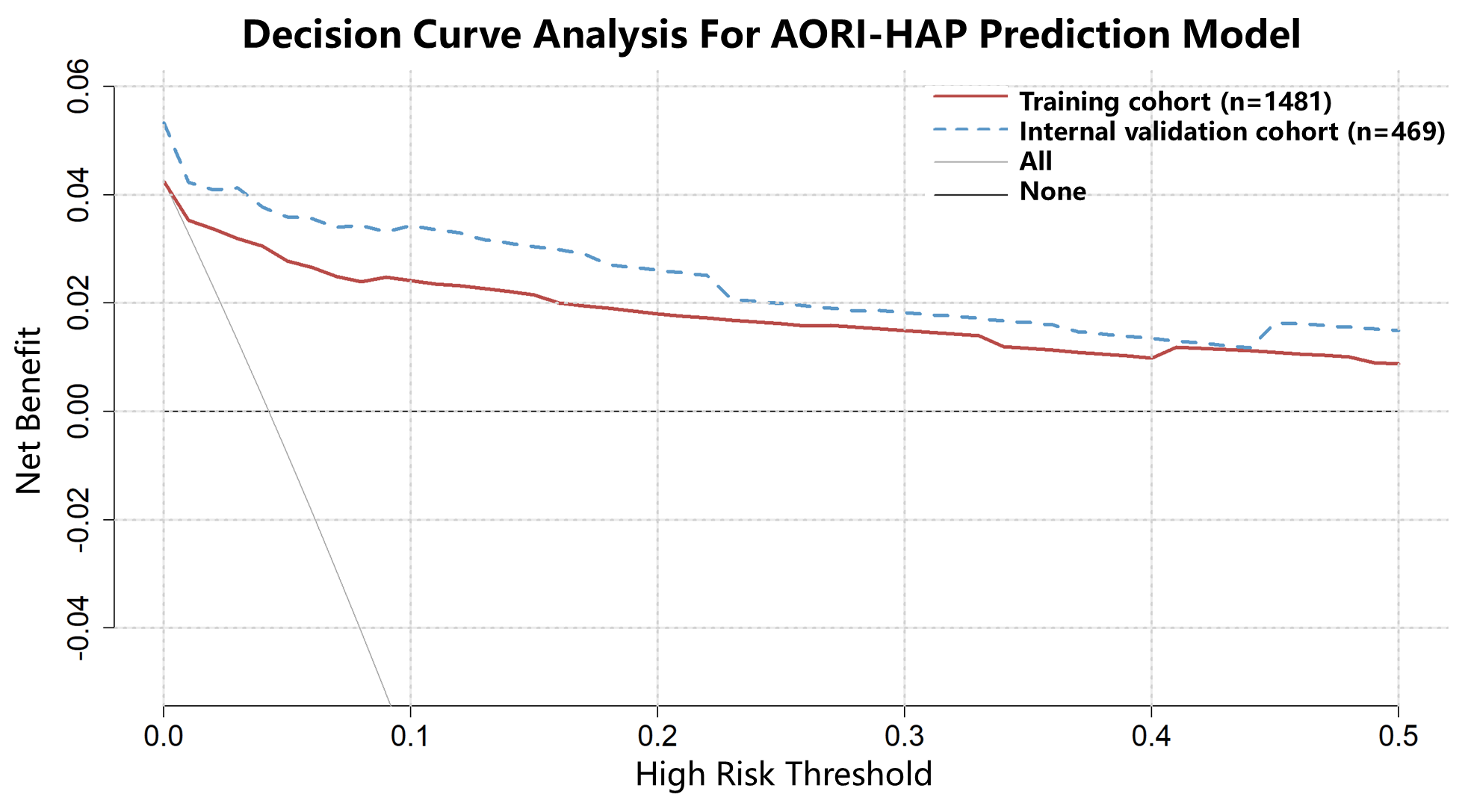


Figure S5 Decision Curve Analysis (DCA) for the AORI-HAP prediction model in hospitalized asthma exacerbation patients. The x-axis represents the threshold probability, and the y-axis indicates the net benefit. The red solid line represents the training cohort (n=1481), and the blue dashed line represents the internal validation cohort (n=469). The “All” and “None” lines correspond to strategies of treating all or no patients, respectively. Across most clinically relevant threshold ranges (0.05–0.45), both training and validation cohorts showed higher net benefit compared with these reference strategies.

**References**

1. Lin S, Ma Y, Zou H. Enhanced Youden's index with net benefit: A feasible approach for optimal-threshold determination in shared decision making. *J Eval Clin Pract*. 2020;26(2):551-558.
2. Fluss R, Faraggi D, Reiser B. Estimation of the Youden Index and its associated cutoff point. *Biom J*. 2005;47(4):458-472.
3. Beijers RJ, van den Borst B, Newman AB, et al. A Multidimensional Risk Score to Predict All-Cause Hospitalization in Community-Dwelling Older Individuals With Obstructive Lung Disease. *J Am Med Dir Assoc*. 2016;17(6):508-513.
4. Wang T, Zhang C, Gao Y, et al. Predictive value of CRP combined with peripheral blood cell ratio for the prognosis of advanced NSCLC. *Am J Cancer Res*. 2023;13(11):5667-5683.
5. Gutin I. In BMI We Trust: Reframing the Body Mass Index as a Measure of Health. *Soc Theory Health*. 2018;16(3):256-271.
6. Jensen GL, Cederholm T, Correia MITD, et al. GLIM Criteria for the Diagnosis of Malnutrition: A Consensus Report From the Global Clinical Nutrition Community. *JPEN J Parenter Enteral Nutr*. 2019;43(1):32-40.
7. Allison SP, Lobo DN. The clinical significance of hypoalbuminaemia. *Clin Nutr*. 2024;43(4):909-914.
8. GBD 2021 Anaemia Collaborators (2023). Prevalence, years lived with disability, and trends in anaemia burden by severity and cause, 1990-2021: findings from the Global Burden of Disease Study 2021. *The Lancet. Haematology*, *10*(9), e713–e734.
9. Pepys MB, Hirschfield GM. C-reactive protein: a critical update [published correction appears in J Clin Invest. 2003 Jul;112(2):299]. *J Clin Invest*. 2003;111(12):1805-1812.
10. Watson J, Round A, Hamilton W. Raised inflammatory markers. *BMJ*. 2012;344:e454.
11. Schuetz P, Christ-Crain M, Thomann R, et al. Effect of procalcitonin-based guidelines vs standard guidelines on antibiotic use in lower respiratory tract infections: the ProHOSP randomized controlled trial. *JAMA*. 2009;302(10):1059-1066.
12. Bouadma L, Luyt CE, Tubach F, et al. Use of procalcitonin to reduce patients' exposure to antibiotics in intensive care units (PRORATA trial): a multicentre randomised controlled trial. *Lancet*. 2010;375(9713):463-474.
13. Crea F. Targeting hypercholesterolaemia: challenges and opportunities. *Eur Heart J*. 2023;44(25):2263-2266.
14. Karr S. Epidemiology and management of hyperlipidemia. *Am J Manag Care*. 2017;23(9 Suppl):S139-S148.
15. Berglund, L., Brunzell, J. D., Goldberg, A. C., Goldberg, I. J., Sacks, F., Murad, M. H., Stalenhoef, A. F., & Endocrine society (2012). Evaluation and treatment of hypertriglyceridemia: an Endocrine Society clinical practice guideline. *The Journal of clinical endocrinology and metabolism*, *97*(9), 2969–2989.
16. Parhofer KG, Laufs U. The Diagnosis and Treatment of Hypertriglyceridemia. *Dtsch Arztebl Int*. 2019;116(49):825-832.
17. Agrawal S, Dhiman RK, Limdi JK. Evaluation of abnormal liver function tests. *Postgrad Med J*. 2016;92(1086):223-234.
18. Newsome PN, Cramb R, Davison SM, et al. Guidelines on the management of abnormal liver blood tests. *Gut*. 2018;67(1):6-19.
19. Tsoris, A., & Marlar, C. A. (2023). Use Of The Child Pugh Score In Liver Disease. In *StatPearls*. StatPearls Publishing
20. Levey AS, Coresh J, Balk E, et al. National Kidney Foundation practice guidelines for chronic kidney disease: evaluation, classification, and stratification [published correction appears in Ann Intern Med. 2003 Oct 7;139(7):605]. *Ann Intern Med*. 2003;139(2):137-147.
21. Thrombosis and Hemostasis Group, Hematology Society of Chinese Medical Association. *Zhonghua Xue Ye Xue Za Zhi*. 2017;38(5):361-363.
22. Schjørring OL, Klitgaard TL, Perner A, et al. Lower or Higher Oxygenation Targets for Acute Hypoxemic Respiratory Failure. *N Engl J Med*. 2021;384(14):1301-1311.
23. Robba C, Battaglini D, Abbas A, et al. Clinical practice and effect of carbon dioxide on outcomes in mechanically ventilated acute brain-injured patients: a secondary analysis of the ENIO study [published correction appears in Intensive Care Med. 2024 Feb;50(2):317-318.
24. American Diabetes Association Professional Practice Committee. 2. Diagnosis and Classification of Diabetes: Standards of Care in Diabetes-2024. *Diabetes Care*. 2024;47(Suppl 1):S20-S42
